# Human Plaque Myofibroblasts to Study Mechanisms of Atherosclerosis

**DOI:** 10.1161/JAHA.123.030243

**Published:** 2023-10-27

**Authors:** Michele F. Buono, Ernest Diez Benavente, Lotte Slenders, Daisey Methorst, Daniëlle Tessels, Eloi Mili, Roxy Finger, Daniek Kapteijn, Mark Daniels, Noortje A. M. van den Dungen, Jorg J. A. Calis, Barend M. Mol, Gert J. de Borst, Dominique P. V. de Kleijn, Gerard Pasterkamp, Hester M. den Ruijter, Michal Mokry

**Affiliations:** ^1^ Laboratory of Experimental Cardiology University Medical Center Utrecht Utrecht the Netherlands; ^2^ Central Diagnostics Laboratory University Medical Center Utrecht Utrecht the Netherlands; ^3^ Department of Cardiology University Medical Center Utrecht Utrecht the Netherlands; ^4^ Center for Translational Immunology University Medical Center Utrecht Utrecht the Netherlands; ^5^ Pediatric Immunology and Rheumatology, Wilhelmina Children’s Hospital University Medical Center Utrecht Utrecht the Netherlands; ^6^ Department of Vascular Surgery University Medical Center Utrecht Utrecht the Netherlands

**Keywords:** disease modeling, myofibroblast, phenotypic modulation, plaque cells, smooth muscle cell, Basic Science Research, Vascular Biology, Atherosclerosis

## Abstract

**Background:**

Plaque myofibroblasts are critical players in the initiation and advancement of atherosclerotic disease. They are involved in the production of extracellular matrix, the formation of the fibrous cap, and the underlying lipidic core via modulation processes in response to different environmental cues. Despite clear phenotypic differences between myofibroblast cells and healthy vascular smooth muscle cells, smooth muscle cells are still widely used as a cellular model in atherosclerotic research.

**Methods and Results:**

Here, we present a conditioned outgrowth method to isolate and culture myofibroblast cells from plaques. We obtained these cells from 27 donors (24 carotid and 3 femoral endarterectomies). We show that they keep their proliferative capacity for 8 passages, are transcriptionally stable, retain donor‐specific gene expression programs, and express extracellular matrix proteins (*FN1*, *COL1A1*, and *DCN*) and smooth muscle cell markers (*ACTA2*, *MYH11*, and *CNN1*). Single‐cell transcriptomics reveals that the cells in culture closely resemble the plaque myofibroblasts. Chromatin immunoprecipitation sequencing shows the presence of histone H3 lysine 4 dimethylation at the *MYH11* promoter, pointing to their smooth muscle cell origin. Finally, we demonstrated that plaque myofibroblasts can be efficiently transduced (>97%) and are capable of taking up oxidized low‐density lipoprotein and undergoing calcification.

**Conclusions:**

In conclusion, we present a method to isolate and culture cells that retain plaque myofibroblast phenotypical and functional capabilities, making them a suitable in vitro model for studying selected mechanisms of atherosclerosis.

**Nonstandard Abbreviations and Acronyms**
GFP: green fluorescent protein
HCASMC: human coronary artery smooth muscle cell
scRNA‐seq: single‐cell RNA sequencing
SMC: smooth muscle cell

**Clinical Perspective**

**What Is New?**

- We established the conditions for an outgrowth method, enabling the isolation of primary plaque myofibroblast cells from human atherosclerotic tissues.
- The isolated cells maintain patient‐specific gene expression, originate from smooth muscle cells, and express myofibroblast markers.

**What Are the Clinical Implications?**

- The use of cultured plaque myofibroblasts offers an exciting opportunity to deepen our comprehension of plaque formation and progression while uncovering new insights into sex‐specific markers of plaque vulnerability.
- These cells provide a valuable in vitro platform to study the effect of drugs on processes involved in atherosclerosis, such as lipid uptake, migration, transdifferentiation, or production of signaling molecules.

Atherosclerosis is a complex systemic condition leading to plaque formation in the arterial walls. Cells with smooth muscle cell (SMC) characteristics play a crucial role in the plaque development as they are responsible for the production of extracellular matrix,^1^ the formation of the fibrous cap, and the underlying lipidic core.^2^ SMCs are likely involved in driving the differences in the cellular composition between plaque phenotypes via modulation processes, in which they transdifferentiate based on different environmental cues.^3^, ^4^, ^5^, ^6^ Regardless of the considerable uncertainty about these modulated SMCs, Wirka et al demonstrated that in atherosclerotic lesions they initially transdifferentiate into a unique fibroblast‐like phenotype in both mouse and human arteries, showing SMC and fibroblast characteristics and acquiring increased plasticity and proliferative and migrative abilities.^5^

Although atherosclerotic plaques constitute several cell types, more than half of them consist of cells derived from SMCs,^2^ which are functionally and phenotypically different. For example, a fraction of SMCs lack detectable expression of some canonical SMC genes, but express mesenchymal (Sca1 and CD105) as well as myofibroblast (α‐smooth muscle actin and platelet‐derived growth factor receptor‐B) markers.^7^ Moreover, lineage‐tracing studies showed that part of plaque SMCs is derived from transdifferentiated cells with non‐SMC origins.^7^, ^8^ Despite the clear phenotypic differences between classic SMCs and the modulated SMCs within atherosclerotic lesions, classic SMCs are still widely used as a cellular model in atherosclerotic research.

Several groups previously attempted to isolate and culture modulated SMCs from plaques by mincing or enzymatically digesting atherosclerotic tissues.^9^, ^10^, ^11^, ^12^, ^13^ However, the explant approach by mincing and culturing the pieces of tissues leads to highly heterogeneous cultures and has a low yield.^9^, ^10^, ^11^ The enzymatic digestion activates the expression of chemokines and adhesion molecules, leading to extensive leukocyte adhesion,^12^ affecting the yield and growth of cells derived from plaque.^10^, ^13^

Here, we present a robust and reproducible conditioned outgrowth explant‐based method for isolating the aforementioned fibroblast‐like cells from human atherosclerotic tissues. These cells closely resemble the morphologic features and the transcriptome of plaque myofibroblast, have SMC origin, and retain patient‐specific gene expression profiles.

## Methods

The data that support the findings of this study are available from the corresponding author on reasonable request.

### Study Design and Participants

Plaque myofibroblasts were isolated from fresh atherosclerotic plaque tissues obtained from patients who underwent carotid or thrombotic (femoral) endarterectomies (CEAs or TEAs, respectively). Written informed consent was obtained from all the patients preoperatively. The study was approved by the Medical Ethical Committee of University Medical Center Utrecht and conducted according to the Declaration of Helsinki.

### Conditioned Outgrow Isolation and Culture of Human Plaque Myofibroblasts

Fresh tissues were collected in Hanks balanced salt solution 1× (catalog number 14025‐50; Gibco) on ice within 30 minutes of operative resection. Tissue fragments were then dissected into 2‐ to 3‐mm^3^ pieces. Then, 1 or 2 pieces were placed in each well of a 12‐well plate, precoated with 2 μg/cm^2^ fibronectin (catalog number F1141; Sigma Aldrich). The pieces were cultured for 14 days, the first 7 days in HAM F12K Complete containing antimicrobial agent for primary cells (Primocin; catalog number ant‐pm2; InvivoGen) (1:500) and then in complete medium only. The plaque pieces were removed from the wells at day 14, and cell colonies were kept in culture. At day 21, the colonies were dissociated and subcultured. Plaque myofibroblasts were cultured by replenishing complete medium one other day and subcultured when 70% to 80% confluent at the density of 5×10^5^ cells per T75 dish. Cell counts were determined using the TC20 (Bio‐Rad Laboratories, Hercules, CA).

### HAM F12K Complete Medium

Complete medium contains: HAM F12K (Kaighn) Nut mix 1× (catalog number 21127 022; Gibco) supplemented with 10% (v/v) heat‐inactivated FCS (Corning), 1% (v/v) penicillin‐streptomycin (10 000 U/mL; catalog number 15140122; Gibco), 1% (v/v) insulin transferrin sodium selenite solution (catalog number 41400045; Fisher Scientific), 10 mmol/L HEPES (catalog number 15630056; Fisher Scientific), 10 mmol/L of N‐[tris(hydroxymethyl)methyl]‐2‐aminoethanesulfonic acid (catalog number T1375; Sigma Aldrich), 30 μg/mL endothelial cell growth factors (catalog number 02‐102; Merck/Sigma Aldrich), and 2.5 μg/mL of vitamin C solution (L‐ascorbic acid; catalog number A4544; Sigma Aldrich). Vitamin C solution was added weekly, freshly made on use.

### FCS‐Depletion Medium

It consists of basal medium HAM F12K (Kaighn) Nut mix 1× supplemented with 2% (v/v) insulin transferrin sodium selenite solution, 20 mmol/L HEPES, 20 mmol/L N‐[tris(hydroxymethyl)methyl]‐2‐aminoethanesulfonic acid, 60 μg/mL endothelial cell growth factors, and 5 μg/mL of vitamin C solution (added weekly and freshly made on use).

### Cell Migration Activity: Scratch Assay

Monolayers of cells were cultivated until they reached 90% to 100% confluency. Subsequently, a “scratch” was induced by mechanically disrupting the cell layer using a pipette tip, resulting in a distinct gap. The cells adjacent to the scratch began to migrate and proliferate to close the wound. To monitor and quantify the migration process, we used the CytoSMART Omni live‐cell analysis platform, which allowed for continuous recording of the scratch area by capturing images every 2 hours. Representative images were acquired at various stages of the healing process, including 0, 6, 12, 18, and 24 hours.

### Immunofluorescence

Plaque myofibroblasts were cultured on fibronectin‐precoated glass cover slips. Then, the cells were fixed with 4% paraformaldehyde (Sigma) for 15 minutes, permeabilized with 0.1% Triton X‐100 in PBS 1× (Gibco) for 10 minutes and blocked with 10% normal goat serum, all at room temperature and with washing steps in between. After, the cells were incubated with Alexa Fluor 488 α‐smooth muscle actin (1:400; Invitrogen; 53‐9760‐82), in 1% bovine serum albumin in PBS (PBSA) at 4 °C overnight. The following day, the slides were washed 3 times with PBS 1× (Gibco) and then incubated with Alexa Fluor 647 Phalloidin (1:400; Invitrogen; A22287) for F‐actin identification, in 1% bovine serum albumin in PBS at room temperature for 60 minutes in the dark. After, the slides were washed 3 times with PBS 1× and incubated with Hoechst (1:10.000) for nuclear staining in PBS 1× for 3 minutes at room temperature in the dark. Glass coverslips were then mounted on microscopy slides (Epredia; J1800AMNZ) using Fluoromount‐G (SouthernBiotech; 0100‐01) and stored at 4 °C for short‐term storage and −20 °C for long‐term storage. Slides were imaged using a fluorescence widefield microscopy system, Olympus BX53.

### Quantitative Polymerase Chain Reaction

Along the cultures, samples of 3×10^5^ plaque myofibroblasts were collected, lysed, and stored at −80 °C in 350 μL of lysis buffer (RA1; catalog number 750961; Macherey‐Nagel) at every passaging step. Total RNA was isolated according to the supplier's protocol (Nucleospin RNA; Macherey‐Nagel). A detailed summary of the mRNA samples per plaque myofibroblast lines can be found in Table S1.

Transcription of 300 ng of DNA‐free RNA into cDNA was performed using the qScript cDNA Synthesis Kit (Quantabio; number 95047). Reverse transcription–quantitative polymerase chain reaction was performed using iQ SYBR Green Supermix (Bio‐Rad) with specific primers in a CFX96 Touch Real‐Time PCR detection system (Bio‐Rad): 5 minutes at 95 °C, followed by 40 cycles of 15 seconds at 95 °C, 30 seconds at specific annealing temperature, and 45 seconds at 72 °C, followed by melting curve analysis to confirm single product amplification. mRNA expression levels were normalized to hHP1BP3 (human heterochromatin protein 1 binding protein 3) reference gene mRNA expression (ΔCt). Relative differences were calculated (ΔΔCt) and presented as fold induction (2^−ΔΔCt^). Primers used are shown in Table S2. Data from 3 different plaque myofibroblast lines were used to assess upregulation/downregulation of canonical SMC markers when compared with commercially available human coronary artery SMCs (HCASMCs).

### Generation of Growth Curves

Cells were subcultured when at 70% to 80% confluence at the density of 5×10^5^ cells per 75 cm^2^ and used from passage 2 until they showed a significant decrease in proliferation. The number of days between 2 subculturing procedures was annotated during the cultures. Data were plotted in GraphPad Prism (V9).

### Flow Cytometry

Dissociated cells were directly transferred to flow cytometry tubes (BD Biosciences) and stained with a live/dead discriminator using 1:1000 Zombie Nir Dye in PBS. Then, the samples were permeabilized using a buffer containing 20% fetal bovine serum, 3% BSA, and 0.2% saponin in PBS for 10 minutes at room temperature and stained for CD14, CD31, CD45, endoglin, VAP1 (vascular adhesion protein 1), CD144, ICAM‐1 (intercellular adhesion molecule 1), E‐selectin, P‐selectin, VCAM‐1 (vascular cell adhesion molecule‐1), CD142, and MCP‐1 (monocyte chemoattractant protein 1) (Table S3) in 1% BSA and 0.1% saponin in PBS in the dark at room temperature for 1 hour. After staining, the samples were resuspended in fluorescence‐activated cell sorting buffer, containing 2% fetal bovine serum, 5 mmol/L EDTA, and 0.01% sodium azide in PBS, and measured by recording 3×10^4^ events using CytoFLEX and Gallios cytometers (Beckman Coulter). The resulting data were analyzed using FlowJo v10 (TreeStar). The antibodies used are reported in Table S3. The details of the analyzed cells are as follow: HCASMCs (PromoCell; C‐12511); human coronary artery endothelial cells (Lonza; CC2585); fibroblasts: human cardiac fibroblasts (PromoCell; C‐12375); peripheral blood mononuclear cells (Mini Donor Service, University Medical Center Utrecht; code: F1P150); and mesenchymal stem cells (Cell Therapy Facility, University Medical Center Utrecht; code: MSC053P3_AL‐MSC071P3_R).

### RNA Sequencing

RNA library preparation was performed, adapting the CEL‐Seq2 protocol for library preparation.^14^, ^15^ The initial reverse transcription reaction primer was designed as follows: an anchored polyT, a unique 6‐bp barcode, a unique molecular identifier of 6 bp, the 5' Illumina adapter, and a T7 promoter. cDNA was used for vitro transcription reaction (AM1334; Thermo‐Fisher). The resulting amplified RNA was fragmented and cleaned. RNA yield and quality were checked by Bioanalyzer (Agilent).

cDNA library construction was initiated according to the manufacturer's protocol, with the addition of randomhexRT primer as random primer. Polymerase chain reaction amplification was performed with Phusion High‐Fidelity PCR Master Mix with HF buffer (NEB) and a unique indexed RNA polymerase chain reaction primer (Illumina) per reaction. Library cDNA yield was checked by Qubit fluorometric quantification (Thermo‐Fisher), and quality was checked by Bioanalyzer (Agilent). Libraries were sequenced on the Illumina Nextseq500 platform with paired‐end, 2×75‐bp system (Utrecht Sequencing Facility).

On sequencing, fastq files were de‐barcoded and split for forward and reverse reads The reads were demultiplexed and aligned to human cDNA reference (Ensembl version 84) using the BWA (0.7.13) by calling “bwa aln” with settings ‐B 6 ‐q 0 ‐n 0.00 ‐k 2 ‐l 200 ‐t 6 for R1 and ‐B 0 ‐q 0 ‐n 0.04 ‐k 2 ‐l 200 ‐t 6 for R2, “bwa sampe” with settings ‐n 100 ‐N 100. Multiple reads mapping to the same gene with the same unique molecular identifier (6‐bp long) were counted as a single read.

### Single‐Cell RNA Sequencing (SPLiT‐Seq)

Passage‐synchronized plaque myofibroblasts were collected at 70% to 80% confluency, counted using the TC20 (Bio‐Rad Laboratories, Hercules, CA) and centrifuged at 350*g* for 6 minutes. The supernatant was removed, and the pellet was rinsed in 1 mL of PBS (pH 7.4) (Gibco). At this stage, the cell suspensions were fixed and processed using the split‐pool ligation‐based transcriptome sequencing (SPLiT‐seq) library preparation method. Briefly, cells were fixed with 1% formaldehyde, permeabilized, and counted. Then, the cells were divided into 8000 cells per well together with reverse transcription mix (Maxima H minus Reverse Transcriptase; ThermoFisher) and incubated for reverse transcription barcoding. Then, the cells were pooled, and divided into DNA barcode plate 1, together with ligation mix, and incubated for 30 minutes at 37 °C. After, blocking solution was added to each well, and the cells were again incubated for 30 minutes at 37 °C. The cells were then collected from plate 1, pooled with addition of extra ligase (T4 DNA ligase; NEB), divided over wells in DNA barcode plate 2, and incubated for 30 minutes at 37 °C. Blocking solution was added to each well, and cells were again incubated for 30 minutes at 37 °C. Then, cells were pooled, lysed, and washed. Sublibraries were made and purified for cDNA with dynabeads+streptavidin; after TemplateSwitch, the cDNA was amplified (with 2×Kapa HiFi hotstart Mastermix; Kapa Biosystems) and size selected via ampure. The cDNA was tagmented, amplicons/libraries were generated with Illumina Nextera XT library preparation kit, and the libraries were bioanalyzed and sequenced.

The SPLiT‐seq protocol was adapted from the study of Rosenberg et al^16^ to include a sample‐specific first barcode for multiplexing. In SPLiT‐seq, reverse transcription and barcode ligation steps occur in fixed intact cells. Cells are pooled and split before the barcoding steps, such that different barcode combinations are introduced to individual cells. Reads with the same barcode combination are analyzed together to determine which transcripts an individual cell expressed.

### Chromatin Immunoprecipitation Sequencing

Chromatin immunoprecipitation was performed using the MAGnify Chromatin Immunoprecipitation System (Thermo Fisher Scientific; 492024), as stated in the manufacturer's manual. In short, the dynabeads were incubated with the antibodies, while in the meantime, cross‐linking of the cells was performed with 1% formaldehyde. The reaction was stopped with 1.25 mol/L glycine. Cells were lysed and sonicated with the S2 sonicator (Covaris) to shear the chromatin into 100‐ to 300‐bp fragments following the program stated in the protocol. The microTUBE AFA Fiber Crimp‐Cap 6×16 mm (Covaris; SKU: 520052) was used with the sonicator. A total of 10 μL of the diluted chromatin was set aside for the input control sample. The diluted chromatin and the dynabeads were added together, and several washing steps were performed, followed by protein digestion using proteinase K. The cross‐linking was reversed at 65 °C, and the samples were purified using the ChIP DNA Clean and Concentrator kit (Zymo Research; D5205). The DynaMagTM‐2 Magnet (Thermo Fisher Scientific; 12321D) was used when working with the magnetic beads.

The immunoprecipitated DNA was used for creating libraries that could then be sequenced. The libraries were created with the NEXTFLEX Rapid DNASeq Kit 2.0 (PerkinElmer; NOVA‐5188‐01) and the NEXTFLEX‐HT Barcodes (PerkinElmer; NOVA‐5188‐12). The DNA in the libraries was visualized using the FlashGel DNA Cassette (Lonza; 57032) together with the gel Loading Buffer II (Invitrogen; AM8546G) and the 50‐bp and 1.5‐Kb FlashGel DNA Marker as ladder (Lonza; 57033). The 2100 Bioanalyzer of Automated Droplet Generator (Agilent) was used to visualize the fragment sizes in each sample. The Qubit 3.0 Fluorometer (Thermo Fisher Scientific; Q33216) together with the Qubit dsDNA HS Assay kit (Thermo Fisher Scientific; Q32854) was used to quantify the DNA concentration of each library. The libraries were pooled and sent to the Utrecht Sequencing Facility. The first run was analyzed using the Illumina NextSeq500 platform with a 1×75‐bp High Output Run type. The last 2 runs were analyzed using the Illumina NextSeq2000 with a 2×50‐bp Run type. Then, the mapped next‐generation sequencing data against the human genome (GRCh37/hg19) were visualized using the Integrative Genomics Viewer.

### Oxidized Low‐Density Lipoprotein Uptake

Plaque myofibroblasts and HCASMCs were seeded (1×10^4^ cells/well) into a black 96‐well plate and maintained in FCS‐depletion medium for 24 hours. After 24 hours, the medium was refreshed and oxidized lipoproteins (oxLDLs; L34357; Thermo Fisher Scientific; 2.5 mg/mL) were added to the cultures. The oxLDLs were diluted to 50, 100, and 200 in FCS‐depletion medium and incubated at 37 °C, 5% CO_2_ for the subsequential 24 hours. After, plaque myofibroblasts were rinsed with 1× PBS (Gibco) and fixed for 15 minutes with 4% paraformaldehyde (Klinipath). Then, they were permeabilizated with 0.1% Triton X‐100 in PBS for 15 minutes on a shaker at room temperature. After 2 washing steps with PBS, the cells were incubated with LipidSpot610 (Biotium; 70069) to stain lipids for 30 minutes on a shaker at room temperature in the dark. Then, they were washed with PBS and incubated with 4′,6‐diamidino‐2‐phenylindole (1:10 000) for 10 minutes on a shaker at room temperature in the dark. After, the cells were washed with PBS and imaged using a widefield microscope with CY5 2.0 filter for lipid visualization (Invitrogen; AMEP4956). The control conditions, cells without any exposure of oxLDL, were used as threshold for the blue (4′,6‐diamidino‐2‐phenylindole) and red (lipids) imaging parameters. The oxLDLs taken up by the cells were quantified with ImageJ using a macro run protocol. Lipid intensities were multiplied for 50, to normalize the values obtained from ImageJ for the cell confluency (50%).

### Osteogenic Stimulation

Osteogenic stimulation of plaque myofibroblasts and HCASMCs was performed in 12‐well plates, seeded in 2 replicate wells per donor in their expansion medium. Osteogenic stimulation was induced 24 hours after seeding using DMEM supplemented with 10% FCS, 1% Glutamax, 1% penicillin/streptomycin, 50 μmol/L L‐ascorbic acid 2‐phosphate sesquimagnesium salt hydrate, 10 mmol/L β‐glycerophosphate disodium salt hydrate, and 0.1 μmol/L water‐soluble dexamethasone (the last 3 all from Sigma‐Aldrich).^17^ The cells were stimulated for 21 days, with osteogenic medium exchange every 3 to 4 days. Alizarin red staining was performed at days 14 and 21, on the cells cultured with expansion control medium or osteogenic medium, to determine deposition of calcified matrix. Briefly, the cells were fixed for 15 minutes in 4% paraformaldehyde, then each well was stained with 500 μL 0.5% Alizarin red (Sigma; in double‐distilled water, pH 4.2) for 10 minutes at room temperature and washed 3 times with double‐distilled water to remove unbound dye. Plates were allowed to dry, and staining was documented microscopically using an EVOS FL Cell Imaging System.

### Transduction

Lentiviral vector containing GFP (green fluorescent protein) reporter gene, phage2‐GFP (Addgene; number 86684), to stably express fluorescent human protein in mammalian cells was propagated in NEB Stable Competent *Escherichia coli* (New England Biolabs). HEK293T/17 cells (ATCC; CRL‐11268) were maintained in antibiotic‐free DMEM supplemented with 10% fetal bovine serum (v/v). On day 1, 3×10^5^ cells per well, resuspended in 4 mL of medium, were seeded in a 6‐well plate. The following day, the antibiotic‐free medium was replaced, 2 mL per well, 2 hours before transfection. Later, the cells in each well were transfected with 1 μg of phage2GFP plasmid, 0.5 μg of pVSV‐G envelope plasmid, 1 μg of pCMV (plasmid encoding viral packaging proteins), and 5 μL of P3000 diluted in 100 μL Opti‐MEM with 7 μL of NaCl. Three days after transfection, the medium was centrifuged at 500*g* for 5 minutes to remove cell debris following filtration using a 0.45‐mm syringe filter. The CMV‐GFP lentiviruses were further concentrated by ultracentrifugation with 20% (w/v) sucrose cushion.

Plaque myofibroblasts were plated for transduction in 6‐well plates (Corning) at the density of 1×10^5^ cells per well and maintained in HAM F12K Complete medium. After 24 hours, CMV‐GFP lentiviruses were added directly to the culture media in each well. At 72 hours after transduction, transduction efficiency was checked via imaging, and flow cytometry was performed to confirm transduction.

### Statistical Analysis

Graphical representation and statistical analysis of quantitative polymerase chain reaction and oxLDL uptake data were obtained using GraphPad Prism V9. Unpaired *t*‐test was used to compare the experimental groups with the control groups. Heat map data are presented as mean of 3 replicates, whereas bar plot data are presented as mean±SD. *P*≤0.05 was deemed statistically significant.

Read counts obtained from RNA‐sequencing data were normalized, and differential gene expression between groups was performed using DESeq2 in R. The screening criteria for differentially expressed genes were adjusted *P*<0.05 and log2 fold change ≥0.5 and ≤−0.5. To generate the volcano plot, the “EnhancedVolcano” package was used. The genes significantly overexpressed are represented by the red dots on the left, whereas the ones significantly underregulated are represented by the red dots on the right. Pathway enrichment analysis was performed using differentially upregulated genes, and the package “clusterProfiler” enriched for “GO_Biological_Process” was used. The SingleR function was run at default settings, using the log‐normalized counts as input.

## Results

### Outgrowth Cultures From Fresh Atherosclerotic Plaques

**Figure 1:**
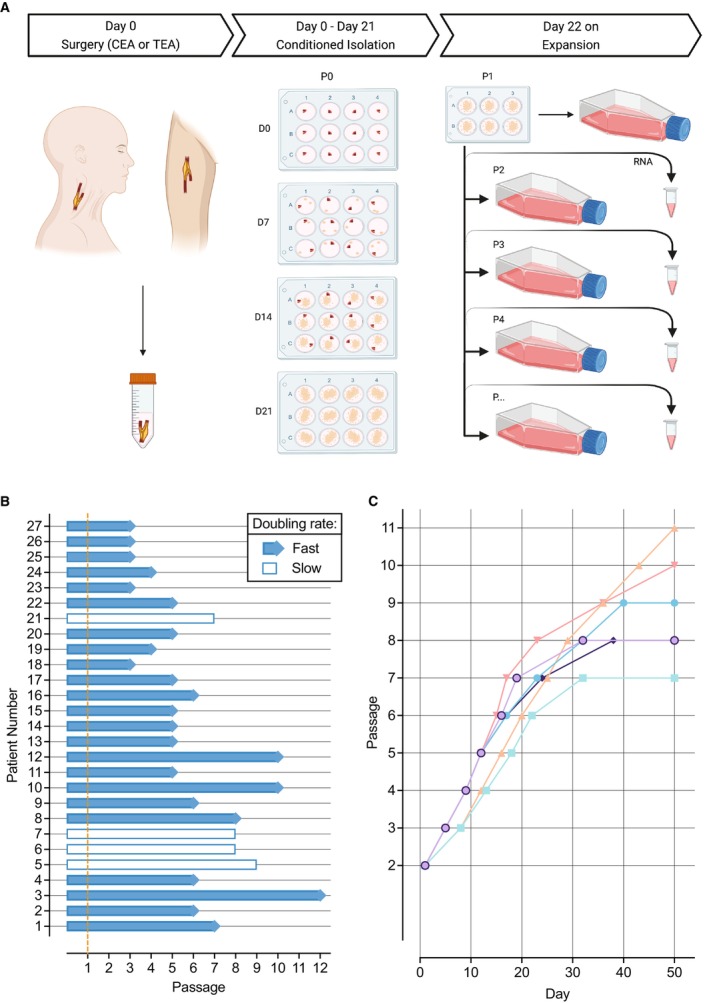
Isolation and growth of plaque myofibroblasts. **A**, Conditioned outgrowth method to isolate and expand plaque myofibroblasts from fresh human atherosclerotic plaques obtained from endarterectomy patients. Plaques were obtained after surgery (D0), cut and placed in a precoated fibronectin 12‐well plate until day 14 (D14), while refreshing the culture medium every other day. At D14, atherosclerotic pieces were removed from the dish, and the cells migrated and clustered in colonies were left to grow until day 21 (D21). On D21, the cell colonies were passed to a 6‐well plate (passage 1 [p1]) and subsequently expanded. From p2 on, plaque myofibroblasts were subcultured until 70% to 80% confluency and sampled for mRNA extraction over the culture. **B**, Growth status of individual plaque myofibroblast lines until their latest passage. The orange dashed line indicates the end of the isolation procedure. **C**, Growth curves of 6 individual examples of plaque myofibroblasts showing their proliferative capacity. CEA indicates carotid endarterectomy; and TEA, thromboendarterectomy.

We tested 2 culture media: IMDM–fetal bovine serum^18^ and HAM F12K Complete (designed in‐house; see Methods), in combination with Matrigel, vitronectin, and fibronectin‐precoated plates, and in the presence and absence of vitamin C. Figure 1 provides an overview of the methods of the conditioned outgrowth method, which is based on the capability of the plaque myofibroblasts to migrate from 2‐ to 3‐mm^2^ plaque pieces (Figure S1A) and proliferate in the dish.^19^, ^20^, ^21^ We compared the culture conditions by observing the cell's growth using transmitted light microscopy (Figure S1B). We evaluated the combination of fibronectin coating, HAM F12K Complete, and vitamin C as optimal for the yield of the cells after 14 days of culture (Figure S1B–S1BN). The addition of vitamin C was crucial to maintain the cell proliferation in culture (Figure S1B through S1N) and has a limited effect on gene expression (Figure S1C). Interestingly, we observed a downregulation of ribosomal activity and an upregulation of adhesion processes (Figure S1D). Moreover, considering that our method primarily focuses on the migratory capacity of cells, we investigated the migrative and proliferative capabilities of plaque myofibroblasts. To assess their migratory potential, we used the scratch assay technique. We found that, within a 24‐hour time frame, plaque myofibroblasts (Figure S2A) exhibited a similar migratory and proliferative trend when compared with HCASMCs (Figure S2B).

We successfully isolated and cultured plaque myofibroblasts from 27 of 31 patients undergoing endarterectomy (Table). The isolation of 4 plaque myofibroblast lines failed because of fungal contaminations. From the 27 atherosclerotic plaques, 24 (11 men and 13 women) were obtained from the carotid artery, and 3 (1 man and 2 women) were obtained from the femoral artery (Table S1).

**Table .** Baseline Characteristics of Patients From Whom Plaque Myofibroblasts Were Isolated

Characteristics	Women	Men	Overall
No. of patients	15	12	27
Age, median (IQR), y	73 (66–80)	77 (70–84)	75 (70–82)
BMI, median (IQR), kg/m^2^	23 (22–26)	25 (24–27)	25 (23–27)
Current smoker, yes, n (%)	4 (27)	4 (33)	8 (30)
Alcohol use, yes, n (%)	8 (57)	8 (67)	16 (62)
Diabetes, yes, n (%)	3 (20)	4 (33)	7 (26)
Hypertension, yes, n (%)	11 (73)	10 (83)	21 (78)
Hypercholesterolemia, yes, n (%)	6 (46)	7 (58)	13 (52)
History of coronary artery disease, n (%)	2 (13)	7 (58)	9 (33)
History of PAOD, n (%)	6 (40)	4 (33)	10 (37)
Use of antiplatelet therapy, n (%)	14 (93)	10 (83)	24 (89)
Use of lipid‐lowering drugs, n (%)	12 (86)	12 (100)	24 (92)
GFR (MDRD equation), mean (SD), mL/min per 1.73 m^2^	86 (21)	70 (21)	79 (22)
LDL, median (IQR), mg/dL	89 (65–105)	75 (69–79)	77 (69–96)
HDL, median (IQR), mg/dL	59 (50–68)	48 (43–50)	51 (46–59)
Total cholesterol, median (IQR), mg/dL	166 (123–178)	151 (148–152)	151 (133–176)
Triglyceride level, median (IQR), mg/dL	137 (117–142)	133 (111–146)	133 (115–142)

BMI indicates body mass index; GFR, glomerular filtration rate; HDL, high‐density lipoprotein; IQR, interquartile range; LDL, low‐density lipoprotein; MDRD, Modification of Diet in Renal Disease; and PAOD, peripheral artery occlusive disease.

We expanded all the isolated plaque myofibroblasts for at least 3 passages (Figure 1B). The cells have an average doubling rate of ≈4 days until passage 8; after that, the proliferative capacity decreases (Figure 1C). Typically, 8 million cells can be obtained from a single isolation by passage 5.

### Characterizing Plaque Myofibroblasts

To characterize plaque myofibroblasts, because of technical limitations, we used cells derived from different passages and donors. We ensured that the cells used were not above passage 8, unless otherwise specified. Further information on the donors and passages used in each experiment can be found in Table S4.

**Figure 2:**
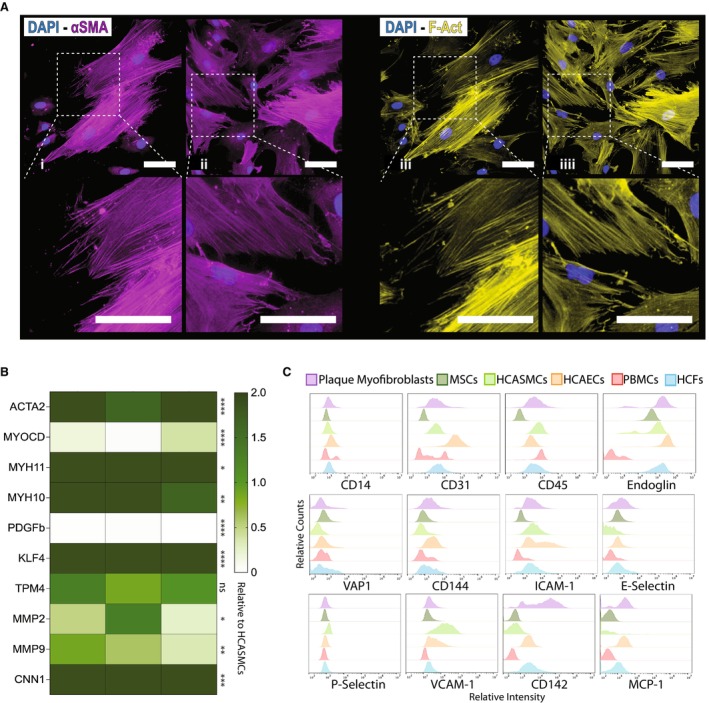
Characterization of plaque myofibroblasts. **A**, Representative immunofluorescence images of plaque myofibroblasts obtained from 2 donors showing positivity for α‐smooth muscle (SM) actin (i–ii) and morphologic tracts via phalloidin staining (F‐actin; iii–iiii). Bars=50 μm. **B**, Gene expression analysis of 3 individual plaque myofibroblast donors for the canonical SM cell (SMC) markers relative to human coronary artery SMCs (HCASMCs). Each column represents 1 patient and the corresponding gene expression pattern. *P≤0.05, **P≤0.01, ***P≤0.001, ****P≤0.0001. **C**, Flow cytometry analysis comparing expression profiles of plaque myofibroblasts with other cell types involved in atherogenesis. The histograms show the “relative counts” on the *y* axis and the “relative intensity” on the *x* axis.

To begin with the characterization of plaque myofibroblasts, we performed immunofluorescence staining, showing that cultured plaque myofibroblasts were positive for α‐smooth muscle actin (Figure 2A, i–ii) and presented a stretched morphologic pattern when stained for phalloidin (F‐actin) (Figure 2A, iii–iiii). On examination of the immunofluorescence images, plaque myofibroblasts displayed a distinct trapezoidal elongated shape, resembling a rectangular‐ or parallelogram‐like structure with elongation along a specific axis. The oval‐shaped nuclei are positioned along the longitudinal cell axis (Figure 2A).

Next, we measured the expression of canonical SMC markers, such as ACTA2 (smooth muscle actin alpha 2), MYOCD (myocardin), MYH11 (myosin heavy chain 11), MYH10 (myosin heavy chain 10), PDGFB (platelet‐derived growth factor subunit B), KLF4 (Krüppel‐like factor 4), TPM4 (tropomyosin 4), MMP2 (matrix metallopeptidase 2), and CNN1 (calponin 1), using quantitative polymerase chain reaction in cultured plaque myofibroblasts and HCASMCs (Table S1). Plaque myofibroblasts had comparable expression levels of all the markers among donors, and they showed lower levels of MYOCD and PDGFB and higher ACTA2, MYH11, KLF4, and CNN1 levels when compared with HCASMCs (Figure 2B).

Flow cytometry was used to compare the protein expression profile of various cell markers in plaque myofibroblasts and other cell types involved in atherogenesis or present in artery walls,^22^ such as HCASMCs, human coronary artery endothelial cells, human cardiac fibroblasts, mesenchymal stem cells, and peripheral blood mononuclear cells. Accordingly, isolated plaque myofibroblasts best matched with SMC and fibroblast profiles but with different expression of markers, like E‐selectin, CD142, and MCP1 (Figure 2C).

### Transcriptional Stability of Plaque Myofibroblasts in Culture

**Figure 3:**
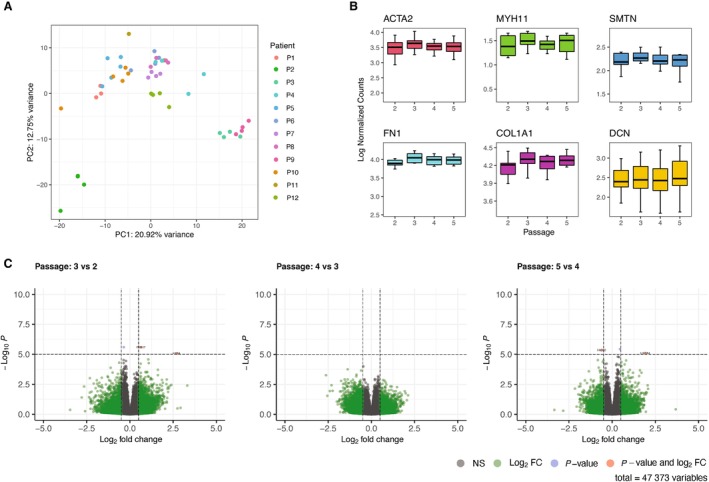
Stable transcriptome maintained over passages. **A**, Principal component analysis, based on the top 500 variable genes, of plaque myofibroblasts from 12 patients. **B**, Box plots showing the expression of canonical smooth muscle cell (SMC) and fibroblast genes, such as *ACTA2*, *MYH11*, *SMTN*, *FN1*, *COL1A1*, and *DCN*, throughout passages (from 2 to 5). **C**, Differential gene expression analysis between passages 2, 3, 4, and 5.

To understand whether the plaque myofibroblasts maintain a stable transcriptome or undergo transcriptional changes in culture, the RNA was extracted from plaque myofibroblasts of 12 individual donors (6 men and 6 women), from passage 2 to 5, and was studied by RNA sequencing (Table S1). Principal component analysis suggests that plaque myofibroblasts of the same donor, at different passages, group together. This demonstrated that the donor characteristics were retained in culture and had higher impact on the cell transcriptome than prolonged culture (Figure 3A).

The expression of canonical SMC and fibroblast markers [ACTA2, MYH11, Smootelin (SMTN), Fibronectin (FN1), Collagen Type I Alpha 1 Chain (COL1A1), and Decorin (DCN)]^23^ was consistent from passage 2 to 5, indicating transcriptome stability (Figure 3B). Using linear models, 316 genes showed a time trend across these passages (Figure S3A). Then, we performed an enrichment analysis that revealed a downregulation of genes associated with proliferation and transcription regulation (Figure S3B). This finding was in line with our previous observation of a decrease in proliferative capacity over time, as shown in Figure 1C. In addition, we performed differential gene expression between consecutive passages. This showed that only 4 coding genes (*P2RX7*, *KIF5A*, *FADS2*, and *LILRA4*) were differentially expressed in independent comparisons from passage 2 to passage 5, suggesting that plaque myofibroblast expression profiles were stable over the passages (Figure 3C). Differential gene expression analysis of passage 1 versus other passages highlighted several immune cell–specific genes (eg, *CD74* and *CD4*) (Figure S3D), and enrichment analysis revealed ongoing inflammatory processes (Figure S3F). In line with this, passage 1 is grouped separately from the higher passages in the principal component analysis plot (Figure S3C). These findings indicated that plaque myofibroblast cultures at passage 1 potentially still contained some cells expressing leukocyte markers, which were strongly reduced from passage 2 onwards.

We observed that plaque myofibroblasts present large and irregularly shaped nuclei as well as a more prominent cytoplasm. These features are consistent in CEA‐ and TEA‐derived plaque myofibroblasts and suggest no differences in the phenotype of these cells (Figure S4A). Plaque myofibroblasts derived from patients undergoing TEA clustered differently compared with those derived from patients undergoing CEA (Figure S4B). Differential gene expression analysis showed transcriptional differences between the cells isolated from carotid and femoral plaques. The TEA‐derived cells overexpressed specific *HOX*‐class genes, such as *HOXC9* (Figure S4C), which is known to play a role in the development and rostrocaudal gene expression patterns.^24^ These findings resonate with findings in dogs, where transcription patterns of vascular cells were dependent on vessel localization.^25^ Moreover, the enrichment analysis showed ongoing pathways related to neutrophils in CEA (Figure S4D). This potentially suggests an increase in inflammation, which aligns with the fact that CEA plaques tend to be more atheromatous compared with TEA plaques, which are commonly more fibrous.^26^

### Transcriptome Analysis Points to the Shared SMC and Fibroblast Characteristics of Isolated Plaque Myofibroblasts

**Figure 4:**
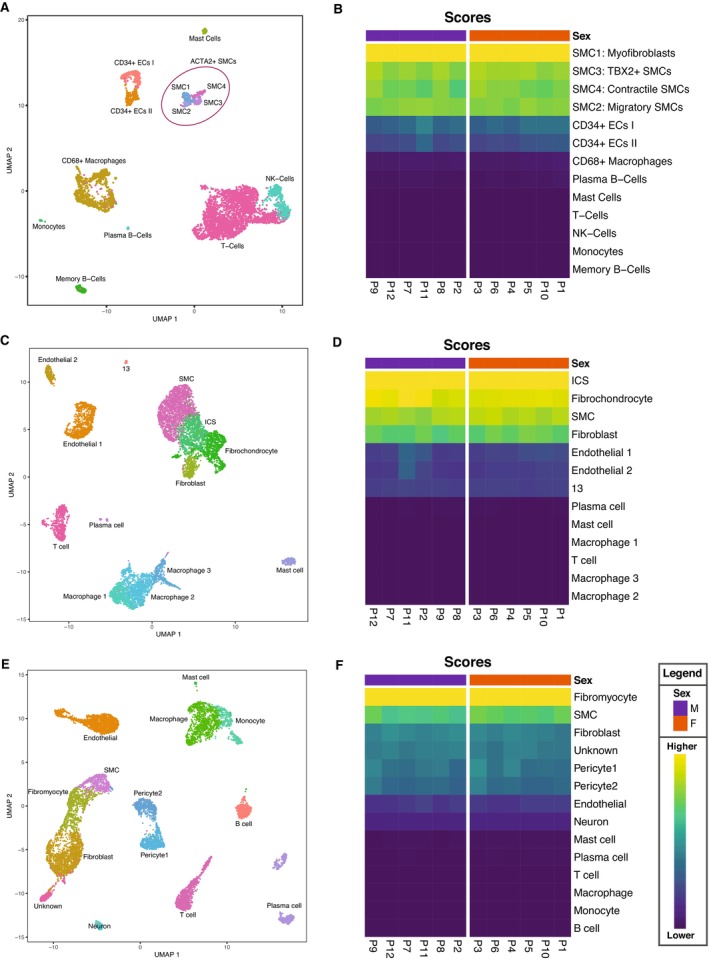
Transcriptome characterization of plaque myofibroblasts. **A**, Uniform manifold approximation and projection (UMAP) showing different cells clusters identified in the single‐cell RNA sequencing (scRNA‐seq) of 46 atherosclerotic plaques of carotid endarterectomy donors, previously generated by our group.^27^ **B**, Heat map showing that plaque myofibroblasts are matched with the ACTA2^+^ smooth muscle cell (SMC) cluster, and specifically with the SMC1 subcluster, which represent cells with myofibroblast characteristics. The *x* axis corresponds to the different patients. **C**, UMAP of scRNA‐seq of 3 atherosclerotic plaques of carotid endarterectomies donors, generated and published by Pan et al.^28^ **D**, Heat map showing that plaque myofibroblasts matched with SMC‐derived intermediate cell state (ICSs), which, according to the authors, is a cluster of cells representing an intermediate state between SMCs and fibroblasts/fibrochondrocytes. The *x* axis corresponds to the different patients. **E**, UMAP of scRNA‐seq of 4 atherosclerotic coronary plaques, generated and published by Wirka et al.^5^ **F**, Heat map showing that plaque myofibroblasts matched with the fibromyocyte cluster, which represents cells with SMC origins and fibroblast characteristics. The *x* axis depicts individual patients.

We projected the RNA‐sequencing data derived from the pooled plaque myofibroblasts from the 12 individual patients onto several existing single‐cell RNA‐sequencing (scRNA‐seq) data sets. The first was generated in our laboratory from 46 human carotid plaques (26 men and 20 women; Figure 4A).^27^ The isolated plaque myofibroblasts most closely resemble ACTA2^+^ SMC transcriptomic profiles (Figure 4A). We found that they best matched with SMC1 cluster (Figure 4B), representing cells with SMC and fibroblast characteristics and hence defined as myofibroblasts.

To validate our finding, we used another human scRNA‐seq carotid plaque data set generated by Pan et al (Figure 4C).^28^ This showed that plaque myofibroblasts mostly match the intermediate cell state cluster (Figure 4D). This cluster represents an intermediate cell state between SMCs and fibroblasts/fibrochondrocytes. We then projected our data on an scRNA‐seq data set of human atherosclerotic coronary arteries (n=4 patients) generated by Wirka et al (Figure 4E).^5^ Interestingly, our plaque myofibroblasts matched fibromyocytes (Figure 4F), which represent modulated cells with an SMC origin and fibroblast characteristics. Although plaque myofibroblasts matched cell clusters named differently among data sets, generated from diverse diseased vascular beds, they all showed the characteristics of an intermediate phenotype between SMCs and fibroblasts.

### scRNA‐Seq Revealed Phenotypic Gradient of Plaque Myofibroblasts

Next, given the diversity of plaque SMCs within atherosclerotic plaques,^29^ we performed scRNA‐seq on plaque myofibroblasts to understand the heterogeneity of the isolated cells. We used a SPLiT‐seq method with passage‐synchronized plaque myofibroblasts from 4 individual patients (2 women and 2 men; passage 4) to compare these with the scRNA‐seq data set of carotid plaque tissue.

**Figure 5:**
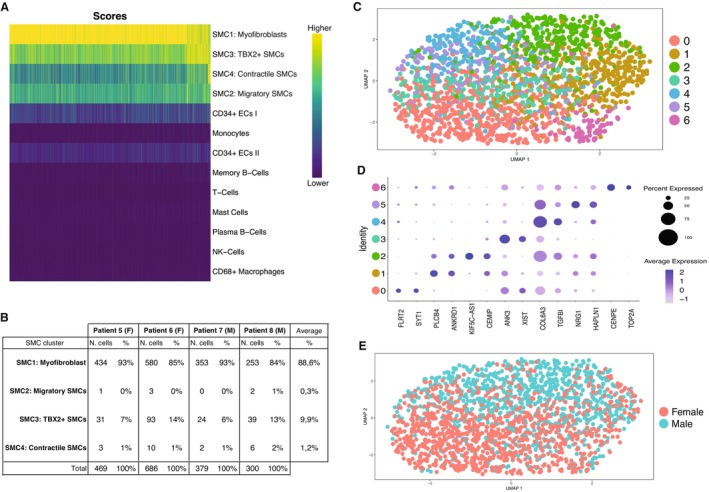
Different subsets of plaque myofibroblasts. **A**, Heat map showing that plaque myofibroblasts matched with the 4 smooth muscle cell (SMC) clusters, mainly with SMC1. **B**, Table showing the distribution of plaque myofibroblasts from 4 individual patients within the 4 SMC clusters. **C**, Uniform manifold approximation and projection (UMAP) generated by plotting plaque myofibroblasts with similar gene expression profiles, showing that 7 different clusters of plaque myofibroblasts can be identified. **D**, The 2 top genes expressed in the 7 clusters were plotted in a dot plot showing differences among plaque myofibroblast cluster profiles. **E**, UMAP of male and female plaque myofibroblasts showing sex‐specific patterns.

A total 1834 cells remained after quality control, which included 469 for patient 5, 686 for patient 6, 379 for patient 7, and 300 for patient 8. These data were then projected to the scRNA‐seq data set from carotid plaques using singleR. Plaque myofibroblasts were distributed among the 4 SMC subclusters, with most matching SMC1 (Figure 5A). On average, plaque myofibroblasts match 88.6% SMC1, 9.9% SMC3, 1.2% SMC4, and 0.3% SMC2 (Figure 5B).

We clustered the cells using Seurat and used uniform manifold approximation and projection (UMAP) to project the individual cells based on their expression similarities. We identified 7 clusters (Figure 5C and 5D), which were not clearly separated in the UMAP (Figure 5D). Interestingly, considering the sexes of the donors, male and female cells were separated in UMAP projection (Figure 5E). We found the *XIST* as one of the genes correlating with this clustering (Figure 5D). Furthermore, sex differential gene expression showed that the sex‐chromosome expression complement of the donor is maintained in vitro (Tables S5 and S6).

### Plaque Myofibroblast Origin

**Figure 6:**
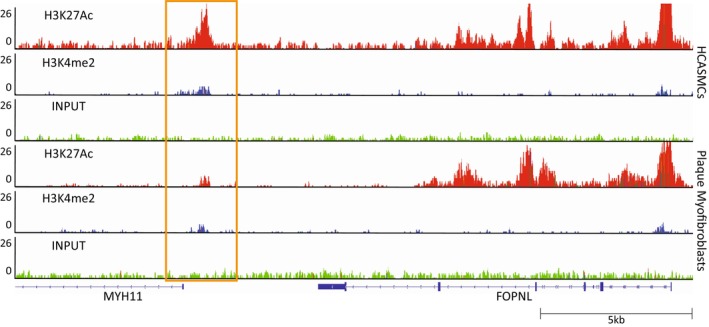
Plaque myofibroblasts have smooth muscle cell (SMC) origin. Chromatin immunoprecipitation sequencing (ChIP‐seq) occupancy of H3K4me3 and H3K27ac in human coronary artery SMCs (HCASMCs) and plaque myofibroblasts at a specific locus of the *MYH11* promoter.

To study the origin of plaque myofibroblasts, we used SMC‐specific epigenetic modification, H3K4me2 on *MYH11* promoter, previously used to trace cells that originate from SMCs.^30^, ^31^ We performed chromatin immunoprecipitation sequencing for the H3K4me2 and H3K27Ac (Figure 6). In both HCASMCs and plaque myofibroblasts, H3K4me2 showed an increased signal compared with the background on the promoter of *MYH11*. On the basis of this finding, the dimethylation mark found in this specific locus of *MYH11* promoter suggests that plaque myofibroblasts have an SMC origin. Moreover, H3K27Ac showed a lower signal in plaque myofibroblasts when compared with HCASMCs, suggesting that *MYH11* promoter is less active in plaque myofibroblasts.

### Plaque Myofibroblasts as a Cellular Model for Atherosclerosis

**Figure 7:**
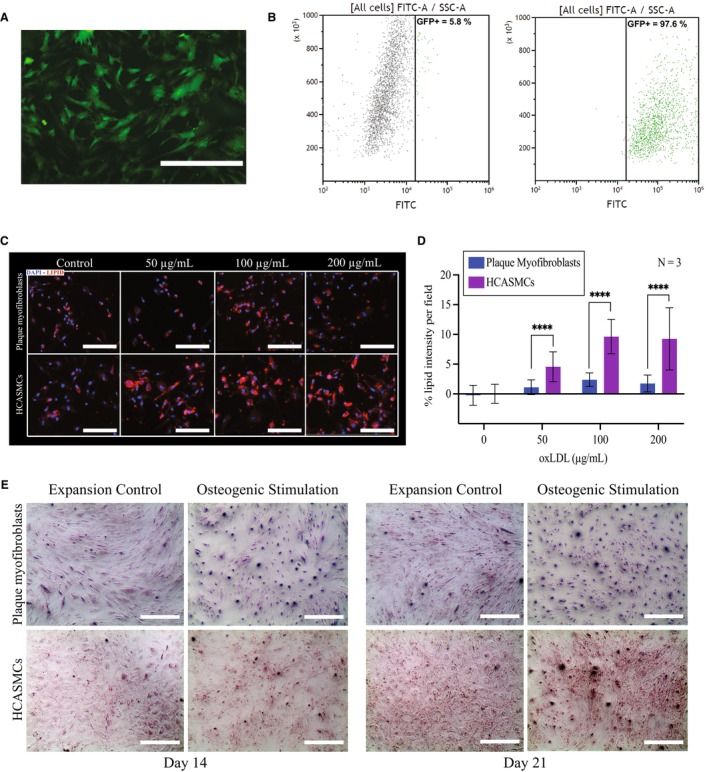
Application of plaque myofibroblasts. **A**, Representative fluorescence image showing GFP (green fluorescent protein)‐positive plaque myofibroblasts (biological replicate=1) 72 hours after lentiviral transduction. Bars=400 μm. **B**, Flow cytometry scatterplots showing GFP‐positive plaque myofibroblasts (biological replicate=2) 72 after transduction. Control nontransduced plaque myofibroblasts are shown in (i), whereas the transduced ones are in (ii). **C**, Representative fluorescence images of plaque myofibroblasts (biological replicate=3) and human coronary artery smooth muscle cells (HCASMCs) (individual technical replicate=3), showing the uptake of oxidized low‐density lipoprotein (oxLDL) (red) after 24 hours of exposure at different concentrations (0, 50, 100, and 200 μg/mL). Bars=200 μm. **D**, Bar plot showing the quantified oxLDL taken up by plaque myofibroblasts and HCASMCs when compared with untreated control (0). Normalized for confluency. ****P≤0.0001. **E**, Representative transmitted light images showing Alazarin red staining to visualize the deposition of calcified matrix in plaque myofibroblasts (biological replicate=1) and HASMCs after 14 and 21 days of osteogenic stimulation and expansion control culture. Bars=400 μm.

Transduction efficiency can be a limiting factor in the usability of cellular models. Therefore, to investigate the transferability of plaque myofibroblasts, we transduced plaque myofibroblasts with CVM‐GFP lentiviruses and showed that most of the cells were GFP‐positive (Figure 7A). Transduction efficiency was measured via flow cytometry, demonstrating that >97% of the cells were expressing GFP and hence successfully transduced (Figure 7B).

Then, we used plaque myofibroblasts as a cellular model for the most important SMC‐related mechanisms involved in the progression of atherosclerosis: oxLDL uptake and calcification. Specifically, we focused on the accumulation of oxLDL, relevant for the formation of the lipid core,^32^ and calcified matrix, leading to vascular calcification and to severe events, such as occlusion of the lumen, plaque rapture, thrombus formation, and infraction.^17^

To check if plaque myofibroblasts were capable of uptake of oxLDL and if there was any difference with commercial SMCs, we exposed plaque myofibroblasts and HCASMCs to different oxLDL concentrations, as 50, 100, and 200 μg/mL. After 24 hours of exposure, nuclei and lipids were stained, and internalized oxLDL was quantified. We showed that both plaque cells and HCASMCs take up oxLDL at all concentrations (Figure 7C). However, plaque myofibroblasts accumulate 4‐fold less oxLDL than HCASMCs (*P*≤0.0001; Figure 7D).

Then, to study the capabilities of plaque myofibroblasts to undergo osteogenic transformations, we osteogenically stimulated plaque myofibroblasts and HCASMCs for 21 days. After, we stained our cells with Alizarin red, which binds to the calcium of calcified matrix. We found the deposition of calcified matrix after 14 days of culture in plaque myofibroblasts, whereas we observed less deposition in HCASMCs. After 21 days of osteogenic stimulation both cell types showed similar deposition of calcified matrix (Figure 7E).

## Discussion

In this study, we established the outgrowth method conditions based on the capability of cells with SMC and fibroblast properties to migrate from the dense structure of atherosclerotic tissue tissues to the culture dish. Although the enrichment analysis did not reveal upregulation of proliferation related pathways, we observed that cells were more viable and exhibited better growth when the medium was supplemented with vitamin C. Interestingly, we found an upregulation of the process involved in cell adhesion, which might explain the positive impact of vitamin C on the cell growth. We reliably and effectively obtained and cultured plaque myofibroblasts from 27 donors, indicating high reproducibility of the isolation protocol. The yield of plaque myofibroblasts and their proliferative activity in vitro are lower when compared with commercial HCASMCs. This is unsurprising, considering that in vivo plaque myofibroblasts are constrained in the dense atherosclerotic tissue, which limits proliferation and the supply of essential nutrition and growth factors. Indeed, atherosclerotic plaques grow over the years before becoming symptomatic or getting diagnosed.^33^ However, 8×10^6^ plaque myofibroblasts can be obtained by passage 5 with a sustained doubling rate of 4 days until passage 8. The possibility to keep them in culture for weeks makes plaque myofibroblasts suitable for the development of several functional tests.

Our transcriptomic studies highlighted that plaque myofibroblasts retain donor characteristics, and these are maintained during prolonged culture. They have a unique expression profile, expressing ACTA2, CNN1, and MYH11, and fibronectin, COL1A1, and DCN, which are accepted as SMC and fibroblast markers, respectively.^23^ The downregulation or absence of MYOCD is considered as the marker that indicates the switch of SMCs toward this more plastic phenotype.^29^ Also, plaque myofibroblasts showed a higher expression of extracellular matrix markers and adhesion protein, such as fibronectin, which suggests their role as the key determinant of plaque structure as connective tissue promotes the formation of the protective fibrous cap.^34^, ^35^

Moreover, differential gene expression indicated that plaque myofibroblasts maintain a stable transcriptome over the culture but also that cells at passage 1 retain inflammatory signals (CD4 and CD74), which may be attributable to leukocyte infiltration. However, from passage 2 onwards, the cultures lose these signals and become more homogeneous. High‐throughput scRNA‐seq showed the presence of 7 subclusters of plaque myofibroblasts that have slightly different expression profiles, which can be related to their plasticity. However, considering the sex of the donors at single‐cell level, female and male cells cluster separately, demonstrating further that donor characteristics are maintained in vitro.

Furthermore, chromatin immunoprecipitation sequencing showed H3K4me2 mark in the *MYH11* promoter, confirming the SMC origin of plaque myofibroblasts. This finding, together with the retained patient‐specific gene expression, makes plaque myofibroblasts suitable for the in vitro characterization of cellular mechanisms involved in plaque progression and for studying functional consequences of atherosclerosis SMC‐specific key driver genes.

During plaque formation and development in vivo, modulated SMCs lose or acquire certain markers, transdifferentiating to different cell types influenced by outer stimuli.^36^, ^37^, ^38^ However, their response may be regulated by the density and the structure of the surrounding connective tissue. Thus, in vitro studies using plaque myofibroblasts may prove useful in exploring their response to external signals and assist in the study of the molecular mechanisms of formation and progression of atherosclerotic lesions. In light of this, we showed that plaque myofibroblasts are capable of taking up oxLDL. This would be useful for future studies to understand how SMC‐derived foam cells originate, which may help improve our understanding of sex‐specific differences in atherosclerotic plaques. Indeed, plaque myofibroblasts from men and women may uptake oxLDL differently, which may drive the process by which women develop plaques richer in extracellular matrix components and with a smaller or absent lipid/necrotic core when compared with men. Moreover, the presence of calcification within atherosclerotic plaques suggests the similarity to chondrocytes, osteocytes, and even osteoclasts.^39^ Calcification is considered a marker of plaque vulnerability and, hence, is more prevalent in plaques developed by men than women.^40^, ^41^ This way, to prove the potential of plaque myofibroblasts and understanding whether they are a key player in determining plaque phenotypes, we performed a calcification study, showing that they were capable of calcification. Furthermore, we also demonstrated that plaque myofibroblasts can easily be transduced, which will open a plethora of new possibilities for further functional tests, like gene silencing or editing studies, of significant therapeutic value in seeking for new gene therapies and druggable targets.

In conclusion, we are aware of all the limitations of cultured cells, especially in that they may differ from the original in vivo phenotype; we also recognize the value in using plaque myofibroblasts to improve our understanding of plaque formation and biology. Although cells from the first passage or directly isolated from plaque may better reflect certain aspects of plaque formation, their use may be limited by low yield and activated stress responses attributable to isolation. Nonetheless, we demonstrated that plaque myofibroblasts retain patient‐specific gene expression, have SMC origin, and express crucial myofibroblast markers, which may inform our knowledge of the original tissue. Cultured plaque myofibroblasts could be used to better study mechanisms affecting plaque formation and progression in a sex‐specific manner but also to search for new markers of plaque vulnerability. Moreover, plaque myofibroblasts may be a good model for in vitro modulation of cell death, regulation of interleukin and adhesion molecule expression, and testing of antiatherosclerotic drugs.

## Sources of Funding

This work has been partly funded with support from the UCARE Horizon 2020 European Research Council Consolidator Grant (866478) to Dr den Ruijter and the Leducq Foundation Transatlantic Network of Excellence (“AtheroGEN”) to Dr den Ruijter.

## Disclosures

None.

## Supporting information

Tables S1–S6
Figures S1–S4

## References

[1] Alexander MR , Owens GK . Epigenetic control of smooth muscle cell differentiation and phenotypic switching in vascular development and disease. Annu Rev Physiol. 2012;74:13–40. doi: 10.1146/annurev-physiol-012110-142315 22017177

[2] Martos‐Rodríguez CJ , Albarrán‐Juárez J , Morales‐Cano D , Caballero A , MacGrogan D , de la Pompa JL , Carramolino L , Bentzon JF . Fibrous caps in atherosclerosis form by Notch‐dependent mechanisms common to arterial media development. Arterioscler Thromb Vasc Biol. 2021;41:e427–e439. doi: 10.1161/ATVBAHA.120.315627 34261328

[3] Newman AAC , Serbulea V , Baylis RA , Shankman LS , Bradley X , Alencar GF , Owsiany K , Deaton RA , Karnewar S , Shamsuzzaman S , et al. Multiple cell types contribute to the atherosclerotic lesion fibrous cap by PDGFRβ and bioenergetic mechanisms. Nat Metab. 2021;3:166–181. doi: 10.1038/s42255-020-00338-8 33619382 PMC7905710

[4] Hartman RJG , Owsiany K , Ma L , Koplev S , Hao K , Slenders L , Civelek M , Mokry M , Kovacic JC , Pasterkamp G , et al. Sex‐stratified gene regulatory networks reveal female key driver genes of atherosclerosis involved in smooth muscle cell phenotype switching. Circulation. 2021;143:713–726. doi: 10.1161/CIRCULATIONAHA.120.051231 33499648 PMC7930467

[5] Wirka RC , Wagh D , Paik DT , Pjanic M , Nguyen T , Miller CL , Kundu R , Nagao M , Coller J , Koyano TK , et al. Atheroprotective roles of smooth muscle cell phenotypic modulation and the TCF21 disease gene as revealed by single‐cell analysis. Nat Med. 2019;25:1280–1289. doi: 10.1038/s41591-019-0512-5 31359001 PMC7274198

[6] Owens GK , Kumar MS , Wamhoff BR . Molecular regulation of vascular smooth muscle cell differentiation in development and disease. Physiol Rev. 2004;84:767–801. doi: 10.1152/physrev.00041.2003 15269336

[7] Shankman LS , Gomez D , Cherepanova OA , Salmon M , Alencar GF , Haskins RM , Swiatlowska P , Newman AAC , Greene ES , Straub AC , et al. KLF4 dependent phenotypic modulation of SMCs plays a key role in atherosclerotic plaque pathogenesis. Nat Med. 2015;21:628–637. doi: 10.1038/nm.3866 25985364 PMC4552085

[8] Alencar GF , Owsiany KM , Karnewar S , Sukhavasi K , Mocci G , Nguyen AT , Williams CM , Shamsuzzaman S , Mokry M , Henderson CA , et al. Stem cell pluripotency genes Klf4 and Oct4 regulate complex SMC phenotypic changes critical in late‐stage atherosclerotic lesion pathogenesis. Circulation. 2020;142:2045–2059. doi: 10.1161/CIRCULATIONAHA.120.046672 32674599 PMC7682794

[9] Bennett MR , Evan GI , Schwartz SM . Apoptosis of human vascular smooth muscle cells derived from normal vessels and coronary atherosclerotic plaques. J Clin Invest. 1995;95:2266–2274. doi: 10.1172/JCI117917 7738191 PMC295839

[10] Dhume AS , Agrawal DK . Inability of vascular smooth muscle cells to proceed beyond S phase of cell cycle, and increased apoptosis in symptomatic carotid artery disease. J Vasc Surg. 2003;38:38–161. doi: 10.1016/s0741-5214(02)75463-3 12844105

[11] Pankajakshan D , Jia G , Pipinos I , Tyndall SH , Agrawal DK . Neuropeptide Y receptors in carotid plaques of symptomatic and asymptomatic patients: effect of inflammatory cytokines. Exp Mol Pathol. 2011;90:280–286. doi: 10.1016/j.yexmp.2011.02.005 21352822 PMC3091977

[12] Koenen RR , Weber C . Chemokines: established and novel targets in atherosclerosis. EMBO Mol Med. 2011;3:713–725. doi: 10.1002/emmm.201100183 22038924 PMC3377113

[13] Bennett S , Breit SN . Variables in the isolation and culture of human monocytes that are of particular relevance to studies of HIV. J Leukoc Biol. 1994;56:236–240. doi: 10.1002/jlb.56.3.236 8083595

[14] Hashimshony T , Wagner F , Sher N , Yanai I . CEL‐Seq: single‐cell RNA‐Seq by multiplexed linear amplification. Cell Rep. 2012;2:666–673. doi: 10.1016/j.celrep.2012.08.003 22939981

[15] Hashimshony T , Senderovich N , Avital G , Klochendler A , de Leeuw Y , Anavy L , Gennert D , Li S , Livak KJ , Rozenblatt‐Rosen O , et al. CEL‐Seq2: sensitive highly‐multiplexed single‐cell RNA‐Seq. Genome Biol. 2016;17:77. doi: 10.1186/s13059-016-0938-8 27121950 PMC4848782

[16] Rosenberg AB , Roco CM , Muscat RA , Kuchina A , Sample P , Yao Z , Graybuck LT , Peeler DJ , Mukherjee S , Chen W , et al. Single‐cell profiling of the developing mouse brain and spinal cord with split‐pool barcoding. Science. 2018;360:176–182. doi: 10.1126/science.aam8999 29545511 PMC7643870

[17] Pustlauk W , Westhoff TH , Claeys L , Roch T , Geißler S , Babel N . Induced osteogenic differentiation of human smooth muscle cells as a model of vascular calcification. Sci Rep. 2020;10:5951. doi: 10.1038/s41598-020-62568-w 32249802 PMC7136202

[18] Novikova OA , Nazarkina ZK , Cherepanova AV , Laktionov PP , Chelobanov BP , Murashov IS , Deev RV , Pokushalov EA , Karpenko AA , Laktionov PP . Isolation, culturing and gene expression profiling of inner mass cells from stable and vulnerable carotid atherosclerotic plaques. PLoS One. 2019;14:e0218892. doi: 10.1371/journal.pone.0218892 31242269 PMC6594632

[19] Pickering JG , Weir L , Rosenfield K , Stetz J , Jekanowski J , Isner JM . Smooth muscle cell outgrowth from human atherosclerotic plaque: implications for the assessment of lesion biology. J Am Coll Cardiol. 1992;20:1430–1439. doi: 10.1016/0735-1097(92)90259-P 1430695

[20] Dartsch PC , Voisard R , Bauriedel G , Höfling B , Betz E . Growth characteristics and cytoskeletal organization of cultured smooth muscle cells from human primary stenosing and restenosing lesions. Arteriosclerosis. 1990;10:62–75. doi: 10.1161/01.ATV.10.1.62 2404491

[21] Libby P , Warner SJ , Salomon RN , Birinyi LK . Production of platelet‐derived growth factor‐like mitogen by smooth‐muscle cells from human atheroma. N Engl J Med. 1988;318:1493–1498. doi: 10.1056/NEJM198806093182303 3367960

[22] Wang D , Wang Z , Zhang L , Wang Y . Roles of cells from the arterial vessel wall in atherosclerosis. Mediat Inflamm. 2017;2017:e8135934. doi: 10.1155/2017/8135934 PMC5478858 28680196

[23] Hu Z , Liu W , Hua X , Chen X , Chang Y , Hu Y , Xu Z , Song J . Single‐cell transcriptomic atlas of different human cardiac arteries identifies cell types associated with vascular physiology. Arterioscler Thromb Vasc Biol. 2021;41:1408–1427. doi: 10.1161/ATVBAHA.120.315373 33626908

[24] Visconti RP , Awgulewitsch A . Topographic patterns of vascular disease: HOX proteins as determining factors? World J Biol Chem. 2015;6:65–70. doi: 10.4331/wjbc.v6.i3.65 26322165 PMC4549770

[25] Spanjersberg TCF , Oosterhoff LA , Kruitwagen HS , van den Dungen NA , Harakalova M , Mokry M , Spee B , van Steenbeek FG . Locational memory of macrovessel vascular cells is transcriptionally imprinted. bioRxiv, 10‐20‐2021. 2021. doi: 10.1101/2021.10.20.465092 PMC10415317 37563195

[26] Herisson F , Heymann M‐F , Chétiveaux M , Charrier C , Battaglia S , Pilet P , Rouillon T , Krempf M , Lemarchand P , Heymann D , et al. Carotid and femoral atherosclerotic plaques show different morphology. Atherosclerosis. 2011;216:348–354. doi: 10.1016/j.atherosclerosis.2011.02.004 21367420

[27] Depuydt MAC , Prange KHM , Slenders L , Örd T , Elbersen D , Boltjes A , de Jager SCA , Asselbergs FW , de Borst GJ , Aavik E , et al. Microanatomy of the human atherosclerotic plaque by single‐cell transcriptomics. Circ Res. 2020;127:1437–1455. doi: 10.1161/CIRCRESAHA.120.316770 32981416 PMC7641189

[28] Pan H , Xue C , Auerbach BJ , Fan J , Bashore AC , Cui J , Yang DY , Trignano SB , Liu W , Shi J , et al. Single‐cell genomics reveals a novel cell state during smooth muscle cell phenotypic switching and potential therapeutic targets for atherosclerosis in mouse and human. Circulation. 2020;142:2060–2075. doi: 10.1161/CIRCULATIONAHA.120.048378 32962412 PMC8104264

[29] Bennett MR , Sinha S , Owens GK . Vascular smooth muscle cells in atherosclerosis. Circ Res. 2016;118:692–702. doi: 10.1161/CIRCRESAHA.115.306361 26892967 PMC4762053

[30] Gomez D , Shankman LS , Nguyen AT , Owens GK . Detection of histone modifications at specific gene loci in single cells in histological sections. Nat Methods. 2013;10:171–177. doi: 10.1038/nmeth.2332 23314172 PMC3560316

[31] Gomez D , Swiatlowska P , Owens GK . Epigenetic control of smooth muscle cell identity and lineage memory. Arterioscler Thromb Vasc Biol. 2015;35:2508–2516. doi: 10.1161/ATVBAHA.115.305044 26449751 PMC4662608

[32] Pirillo A , Norata GD , Catapano AL . LOX‐1, OxLDL, and atherosclerosis. Mediators Inflamm. 2013;2013:152786. doi: 10.1155/2013/152786 23935243 PMC3723318

[33] van Gils MJ , Vukadinovic D , van Dijk AC , Dippel DWJ , Niessen WJ , van der Lugt A . Carotid atherosclerotic plaque progression and change in plaque composition over time: a 5‐year follow‐up study using serial CT angiography. AJNR Am J Neuroradiol. 2012;33:1267–1273. doi: 10.3174/ajnr.A2970 22345501 PMC7965515

[34] Rohwedder I , Montanez E , Beckmann K , Bengtsson E , Dunér P , Nilsson J , Soehnlein O , Fässler R . Plasma fibronectin deficiency impedes atherosclerosis progression and fibrous cap formation. EMBO Mol Med. 2012;4:564–576. doi: 10.1002/emmm.201200237 22514136 PMC3407945

[35] Moore KJ , Fisher EA . The double‐edged sword of fibronectin in atherosclerosis. EMBO Mol Med. 2012;4:561–563. doi: 10.1002/emmm.201200238 22649036 PMC3407944

[36] Xie C , Ritchie RP , Huang H , Zhang J , Chen YE . Smooth muscle cell differentiation in vitro: models and underlying molecular mechanisms. Arterioscler Thromb Vasc Biol. 2011;31:1485–1494. doi: 10.1161/ATVBAHA.110.221101 21677291 PMC3123451

[37] Tabas I , García‐Cardeña G , Owens GK . Recent insights into the cellular biology of atherosclerosis. J Cell Biol. 2015;209:13–22. doi: 10.1083/jcb.201412052 25869663 PMC4395483

[38] Feil S , Fehrenbacher B , Lukowski R , Essmann F , Schulze‐Osthoff K , Schaller M , Feil R . Transdifferentiation of vascular smooth muscle cells to macrophage‐like cells during atherogenesis. Circ Res. 2014;115:662–667. doi: 10.1161/CIRCRESAHA.115.304634 25070003

[39] Qiao J‐H , Mishra V , Fishbein MC , Sinha SK , Rajavashisth TB . Multinucleated giant cells in atherosclerotic plaques of human carotid arteries: identification of osteoclast‐like cells and their specific proteins in artery wall. Exp Mol Pathol. 2015;99:654–662. doi: 10.1016/j.yexmp.2015.11.010 26551087

[40] Alexopoulos N , Raggi P . Calcification in atherosclerosis. Nat Rev Cardiol. 2009;6:681–688. doi: 10.1038/nrcardio.2009.165 19786983

[41] de Bakker M , Timmerman N , van Koeverden ID , de Kleijn DPV , de Borst GJ , Pasterkamp G , Boersma E , den Ruijter HM . The age‐ and sex‐specific composition of atherosclerotic plaques in vascular surgery patients. Atherosclerosis. 2020;310:1–10. doi: 10.1016/j.atherosclerosis.2020.07.016 32861960

