# Supplementary material for: Human Plaque Myofibroblasts to Study Mechanisms of Atherosclerosis
Source: J Am Heart Assoc. 2023 Oct 27;12(21):e030243. doi: 10.1161/JAHA.123.030243 (PMC10727388; doi:10.1161/JAHA.123.030243)
Supplement: Supplementary file 1 — Tables S1–S6 Figures S1–S4 [file JAH3-12-e030243-s001.pdf]

Supplemental Material

Table S1. Patients used for plaque myofibroblast isolation, cells bio-banked and mRNA samples isolated along the cultures.

Patient	Sex	Type	Application	Bio-banked	Last passage Bio-banked	mRNA Samples (Passage)									
						P 1	P 2	P 3	P 4	P 5	P 6	P 7	P 8	P 9	P 10
1	F	CEA	IF, FC, RNA-seq	13x10^{6}	7		✓	✓	✓	✓					
2	M	CEA	IF, FC, RNA-seq	5x10^{6}	6		✓	✓	✓	✓					
3	F	TEA	qPCR, RNA-seq, oxLDL	30x10^{6}	11		✓	✓	✓	✓	✓	✓	✓	✓	✓
4	F	CEA	qPCR, RNA-seq, oxLDL	6.5x10^{6}	6		✓	✓	✓	✓					
5	F	CEA	qPCR, RNA-seq, scSeq, ChIP-seq, oxLDL	15x10^{6}	9	✓	✓	✓	✓	✓	✓	✓	✓	✓	✓
6	F	CEA	RNA-seq, scSeq	10x10^{6}	8		✓	✓	✓	✓	✓	✓	✓	✓	
7	M	CEA	RNA-seq, scSeq	4x10^{6}	6		✓	✓	✓	✓	✓	✓	✓	✓	
8	M	CEA	RNA-seq, scSeq, ChIP-seq, Transduction	10x10^{6}	8		✓	✓	✓	✓					
9	M	TEA	RNA-seq	6x10^{6}	6		✓	✓	✓	✓					
10	F	CEA	RNA-seq, Osteogenic	4x10^{6}	5	✓	✓	✓	✓	✓					
11	M	CEA	RNA-seq	1x 10^{6}	4			✓	✓						
12	M	CEA	RNA-seq	3.5x10^{6}	5		✓	✓	✓	✓	✓	✓	✓	✓	✓
13	M	CEA	-	7x10^{6}	5		✓	✓	✓	✓					
14	M	CEA	-	9x10^{6}	4		✓	✓	✓	✓					
15	F	CEA	-	5x10^{6}	5										
16	M	CEA	-	4x10^{6}	6	✓	✓	✓	✓	✓	✓				
17	M	CEA	-	5x10^{6}	5	✓	✓	✓	✓						
18	M	CEA	-	5x10^{6}	5	✓	✓								
19	F	CEA	-	4x10^{6}	5			✓	✓						
20	F	CEA	-	4x10^{6}	5	✓	✓	✓	✓	✓					
21	F	CEA	-	4x10^{6}	3		✓	✓	✓	✓	✓				
22	F	CEA	-	3.5x10^{6}	5										
23	M	CEA	-	4.5x10^{6}	3										
24	F	CEA	-	4x10^{6}	3		✓	✓	✓						
25	F	CEA	-	1x10^{6}	3										
26	F	CEA	-	2.5x10^{6}	3										
27	F	TEA	-	2x10^{6}	3										

CEA = Carotid Endarectomy: TEA = Thromboendarectomy; IF = Immunofluorescence; FC = Flow Cytometry; RNA-seq = RNA sequencing; scSeq = single cell RNA sequencing; ChIP-seq = Chromatin Immunoprecipitation sequencing.

Table S2. Primers used for qPCR.

Gene Name	Forward Primer	Reverse Primer	Amplicone (bp)	GeneID NCBI	Annealing T. (°C)
hHP1BP3	CCCACGTCCCAAGATGGAT	CTGATGCACCACTCTTCTGGAA	71	50809	60.5 – 62.6
ACTA2	CTATGAGGGCTATGCCTTGCC	GCTCAGCAGTAGTAACGAAGGA	122	59	60.5
MYOCD	CCACCTATGGACTCAGCCTAC	CTCAGTGGCGTTGAAGAAGAG	188	93649	62.6
MYH11	CGCCAAGAGACTCGTCTGG	TCTTTCCCAACCGTGACCTTC	129	4629	62.6
MYH10	GCAGGAGAACACCTAAAGTCTG	TGTCCCGGAATAGGAATATAGCC	89	4628	62.6
PDFGB	CTCGATCCGCTCCTTTGATGA	CGTTGGTGCGGTCTATGAG	239	5155	60.5
KLF4	CCCACATGAAGCGACTTCCC	CAGGTCCAGGAGATCGTTGAA	170	9314	62.6
H-CAD	TCGACCCATCAAGCAGCAATA	CCGGCTTTGTAGGTTTTGCG	100	800	62.5
CNN1	CTGTCAGCCGAGGTTAAGAAC	GAGGCCGTCCATGAAGTTGTT	123	1264	62,6
TAGLN-2	AGTGCAGTCCAAAATCGAGAAG	CTTGCTCAGAATCACGCCAT	154	6876	60.5
MMP2	GATACCCCTTTGACGGTAAGGA	CCTTCTCCCAAGGTCCATAGC	112	4313	60.5
MMP9	TGTACCGCTATGGTTACACTCG	GGCAGGGACAGTTGCTTCT	97	4318	60.5
TPM4	AATTTGCAGAGAGAACGGTTGC	CAGTGTCTGATGTAAGCCCAC	104	7171	60.5

Table S3. Antibodies used for flow cytometry.

Primary Antibody	Fluor-chrome	Clone	D. F.	Cat. N.	Supplier
Zombie NIR	APC-eFluor780	-	1:200	423105	Biolegend
CD14	BV785	HCD14	1:200	325628	Biolegend
CD31	BV605	WM59	1:200	303121	Biolegend
CD45	PerCP/Cy5.5	2D1	1:200	368504	Biolegend
Endoglin (CD105)	Pacific Blue - BV421	SN6h	1:200	800510	Biolegend
VAP1 (AOC3)	APC -AF647	393106	1:200	IC39571R	R&D System
CD144	PE/Cy7	BV9	1:200	348506	Biolegend
ICAM-1 (CD54)	AF488	HCD54	1:200	322713	Biolegend
E-Selectin (CD62E)	PE/Cy7	HAE-1f	1:200	336015	Biolegend
P-Selectin (CD62P)	BV785	AK4	1:200	304941	Biolegend
VCAM-1 (CD106)	BV421	STA	1:200	305815	Biolegend
CD142	PE	NY2	1:200	365203	Biolegend
MCP-1	APC	5D3-F7	1:200	502611	Biolegend

Table S4. Patients and passages used for each experiment.

Application	Figure n.	Patient n.	Passage used	Biological replicates
Immunofluorescence	2 A	1 – 2	2 – 3	2
qPCR	2 B	3 – 4 – 5	3 – 4 – 3	3
Flow Cytometry	2 C	1 – 2	4 – 3	2
RNA-seq	3 – 4	1 to 12	1 to 5	12
sc-Seq	5	6 – 7 – 8	4	3
ChIP-seq	6	5	8	1
Transduction	7 A	8	3	1
oxLDL	7 B	3 – 4 – 5	5 – 5 – 4	3
Osteogenic	7 C	10	6	1

Table S5. Overexpressed genes in male plaque myofibroblasts after sex differential gene expression.

	baseMean	log2FoldChange	lfcSE	stat	pvalue	padj	BH
**RPS4Y1**	83,38306667	6,283552084	0,22756129	27,61257015	7,86E-168	8,21E-164	1,72E-163
**DDX3Y**	44,06423751	7,75982845	0,378998572	20,47455855	3,63E-93	2,53E-89	5,28E-89
**TXLNGY**	13,47171618	4,124143446	0,23227425	17,75549138	1,56E-70	8,16E-67	1,71E-66
**PRKY**	26,58350095	2,497117531	0,156685798	15,93710191	3,50E-57	1,46E-53	3,06E-53
**ZFY**	10,24839925	4,203983833	0,265966108	15,80646445	2,81E-56	9,78E-53	2,04E-52
**KDM5D**	10,04652947	3,013213158	0,210120779	14,3403864	1,22E-46	3,65E-43	7,63E-43
**USP9Y**	10,90284427	2,620427292	0,183872107	14,25135837	4,40E-46	1,15E-42	2,40E-42
**NLGN4Y**	10,03166536	3,991478408	0,286199777	13,94647632	3,31E-44	7,67E-41	1,60E-40
**TTTY14**	7,763295944	5,066442963	0,381960202	13,2643216	3,73E-40	7,79E-37	1,63E-36
**ENSG00000233864**	32,34671638	1,26868508	0,115036471	11,02854657	2,78E-28	4,85E-25	1,01E-24
**ENSG00000266891**	14,7886604	5,784515451	0,61400553	9,420950081	4,47E-21	6,67E-18	1,39E-17
**EIF1AY**	25,59142535	1,217382773	0,131198979	9,278904342	1,71E-20	2,39E-17	4,98E-17
**DUXAP8**	57,10231725	1,536278879	0,203758368	7,539709393	4,71E-14	5,18E-11	1,03E-10
**DUXAP10**	55,26907006	1,658917753	0,242519469	6,840348788	7,90E-12	7,50E-09	1,50E-08
**ENSG00000279508**	22,68280711	2,113567895	0,325715132	6,489007379	8,64E-11	7,04E-08	1,42E-07
**UTY**	6,062000805	1,271327831	0,195985864	6,486834315	8,77E-11	7,04E-08	1,42E-07
**SPESP1**	2,893142195	2,300927263	0,39577951	5,81365938	6,11E-09	4,12E-06	8,34E-06
**COL6A3**	428,1799093	0,800880225	0,143588249	5,577616781	2,44E-08	1,59E-05	3,23E-05
**LRRC15**	11,71593525	1,76796779	0,322935174	5,474683257	4,38E-08	2,69E-05	5,47E-05
**ENSG00000272872**	15,22556393	1,75920323	0,322766848	5,450383889	5,03E-08	2,92E-05	5,93E-05
**PAX8-AS1**	46,82978001	1,124260843	0,206271881	5,45038344	5,03E-08	2,92E-05	5,93E-05
**H19**	34,29625623	3,048481672	0,598836003	5,090678682	3,57E-07	0,000196175	0,000399379
**GPR153**	23,33516141	0,904848572	0,179025227	5,054307627	4,32E-07	0,000231417	0,000471436
**TUSC3**	61,21434231	1,230403801	0,245625261	5,009272236	5,46E-07	0,000278432	0,000567905
**TENM2**	5,991985079	2,567211553	0,517351292	4,962221207	6,97E-07	0,000338636	0,000691467
**HS3ST3A1**	7,261744705	2,498838812	0,512446567	4,876291443	1,08E-06	0,000480557	0,000983159
**GFPT2**	39,12166794	1,03398169	0,216624974	4,773141679	1,81E-06	0,000715025	0,001466309
**CEACAM19**	85,61238922	0,587991883	0,124736621	4,713867344	2,43E-06	0,00090687	0,001861576
**LTBP1**	674,6297929	0,583182412	0,124473	4,685212152	2,80E-06	0,001025163	0,002105048
**ZP3**	22,39820488	0,697014964	0,149944416	4,648488982	3,34E-06	0,001204559	0,002474152
**SPON2**	12,65664837	1,09100789	0,239070021	4,56354956	5,03E-06	0,001694973	0,003485268
**ENSG00000274877**	14,72823891	0,863253124	0,192136625	4,49291292	7,03E-06	0,002225168	0,004579882
**ENSG00000259869**	13,84651242	1,635175395	0,365381868	4,475250519	7,63E-06	0,0023801	0,004899858
**PDGFA**	157,2699769	0,565486927	0,126875416	4,457025197	8,31E-06	0,002516511	0,005182893
**LINC00942**	4,324032776	1,287119665	0,290723239	4,427302302	9,54E-06	0,00284812	0,005867056

Table S6. Overexpressed genes in female plaque myofibroblasts after sex differential gene expression.

	baseMean	log2FoldChange	lfcSE	stat	pvalue	padj	BH
**XIST**	111,966434	-5,635394	0,18118504	-31,102976	2,20E-212	4,59E-208	9,58E-208
**KDM5C**	98,9524396	-0,6099857	0,05142784	-11,861	1,89E-32	3,58E-29	7,49E-29
**PUDP**	31,9847779	-1,0863492	0,12559102	-8,649895	5,15E-18	6,73E-15	1,41E-14
**TSIX**	148,523819	-0,7501758	0,09184684	-8,1676822	3,14E-16	3,86E-13	8,07E-13
**OFD1**	32,907764	-0,782331	0,09827168	-7,9608998	1,71E-15	1,98E-12	3,92E-12
**RPS4X**	1274,02182	-0,5656642	0,07561463	-7,4808838	7,38E-14	7,71E-11	1,53E-10
**ZRSR2**	25,2651395	-0,7639271	0,1107962	-6,8948849	5,39E-12	5,36E-09	1,07E-08
**DHRS9**	45,4017514	-2,2295294	0,32799564	-6,7974361	1,06E-11	9,67E-09	1,94E-08
**GEMIN8**	10,547866	-0,8556854	0,1465671	-5,8381817	5,28E-09	3,70E-06	7,49E-06
**CA5BP1**	78,6613873	-0,5906688	0,10119579	-5,8368908	5,32E-09	3,70E-06	7,49E-06
**SUCNR1**	17,3236946	-2,7980761	0,50241966	-5,569201	2,56E-08	1,62E-05	3,29E-05
**TRPC4AP**	76,7868846	-0,5252915	0,10439897	-5,0315776	4,86E-07	0,0002541	0,00051797
**EHD3**	60,9610731	-0,6023846	0,12067191	-4,9919207	5,98E-07	0,0002974	0,00060694
**RTN1**	21,46786	-2,7666473	0,56612966	-4,8869498	1,02E-06	0,0004755	0,00097192
**IL33**	7,39444364	-2,4147178	0,50292321	-4,8013648	1,58E-06	0,00068597	0,00140401
**LIPA**	267,460358	-0,5197837	0,10835207	-4,7971738	1,61E-06	0,00068618	0,00140502
**RPS4XP6**	17,6981069	-0,5854776	0,1222421	-4,7894924	1,67E-06	0,00069666	0,00142761
**GSTT2**	6,3718031	-1,5386258	0,32177965	-4,781613	1,74E-06	0,00069872	0,00143236
**NES**	66,0243542	-1,1343093	0,2388671	-4,7487048	2,05E-06	0,00079213	0,00162498
**KIAA1549L**	26,5581362	-0,7413372	0,16024708	-4,6262134	3,72E-06	0,00131884	0,00270967
**RPS4XP11**	50,5848409	-0,5044443	0,10941216	-4,6104955	4,02E-06	0,00137596	0,00282856
**SLC16A14**	7,48290265	-3,6815184	0,81441713	-4,5204334	6,17E-06	0,00204672	0,00420961

Figure S1. Establishment of the isolation method.

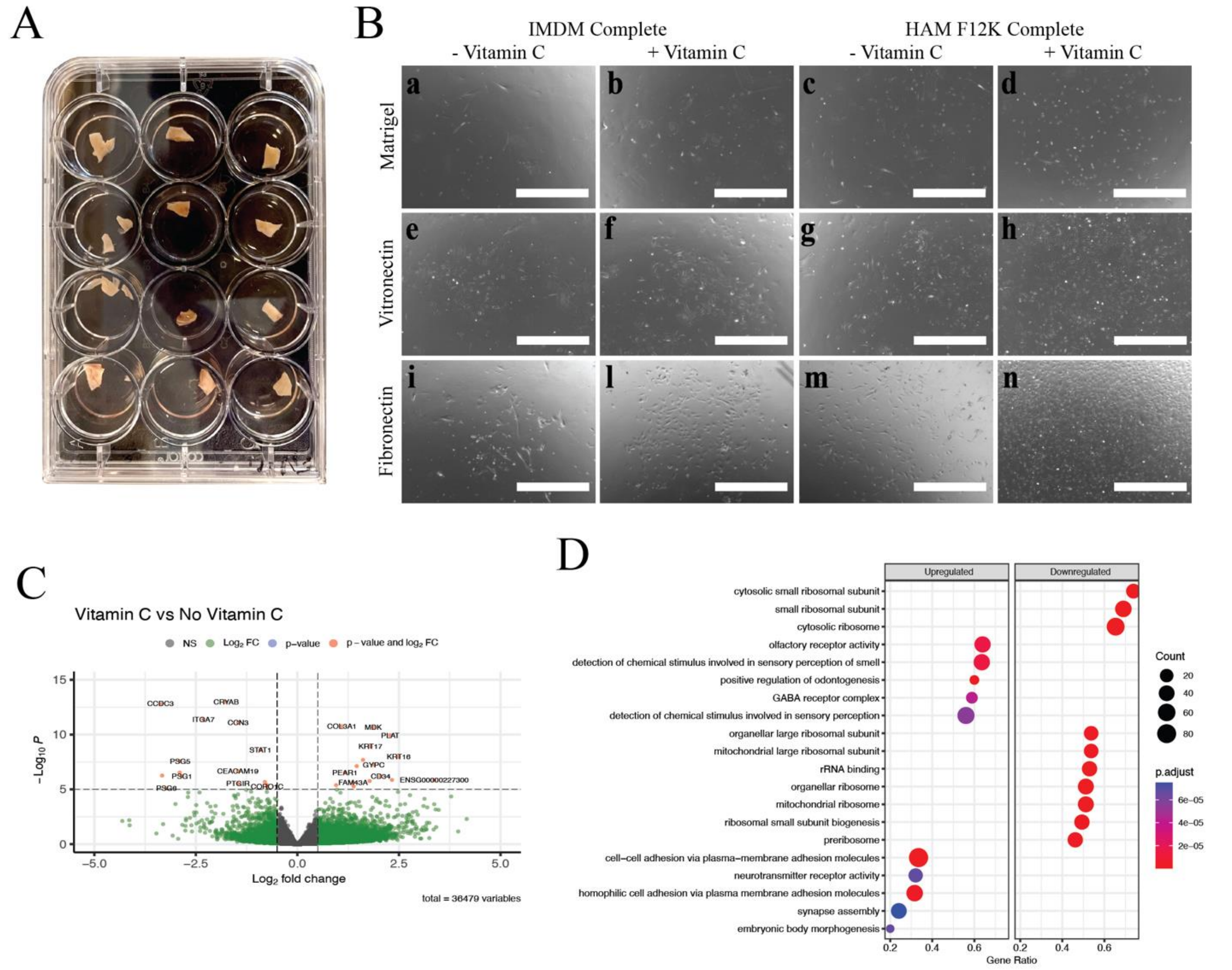

(A) Representative picture of a 12 well-plate containing the 2-3 mm^2 plaque pieces during cell isolation. **(B)** Representative transmitted light images taken after keeping the plaque pieces in culture for 14 days. Two different culture media were tested (IMDM-FCS and HAM F12K Complete), with and without the addition of Vitamin C, in combination with three coating materials (Matrigel, Vitronectin and Fibronectin). Scale bars = 400 µm **(C)** Volcano Plot showing differentially expressed gene in plaque myofibroblasts cultured in HAM F12K (Fibronectin) with and without Vitamin C. **(D)** GO pathway enrichment analysis of DEGs in plaque myofibroblasts cultured in HAM F12K (Fibronectin) with versus without Vitamin C.

Figure S2. Migration activity of plaque myofibroblasts and HCASMCs.

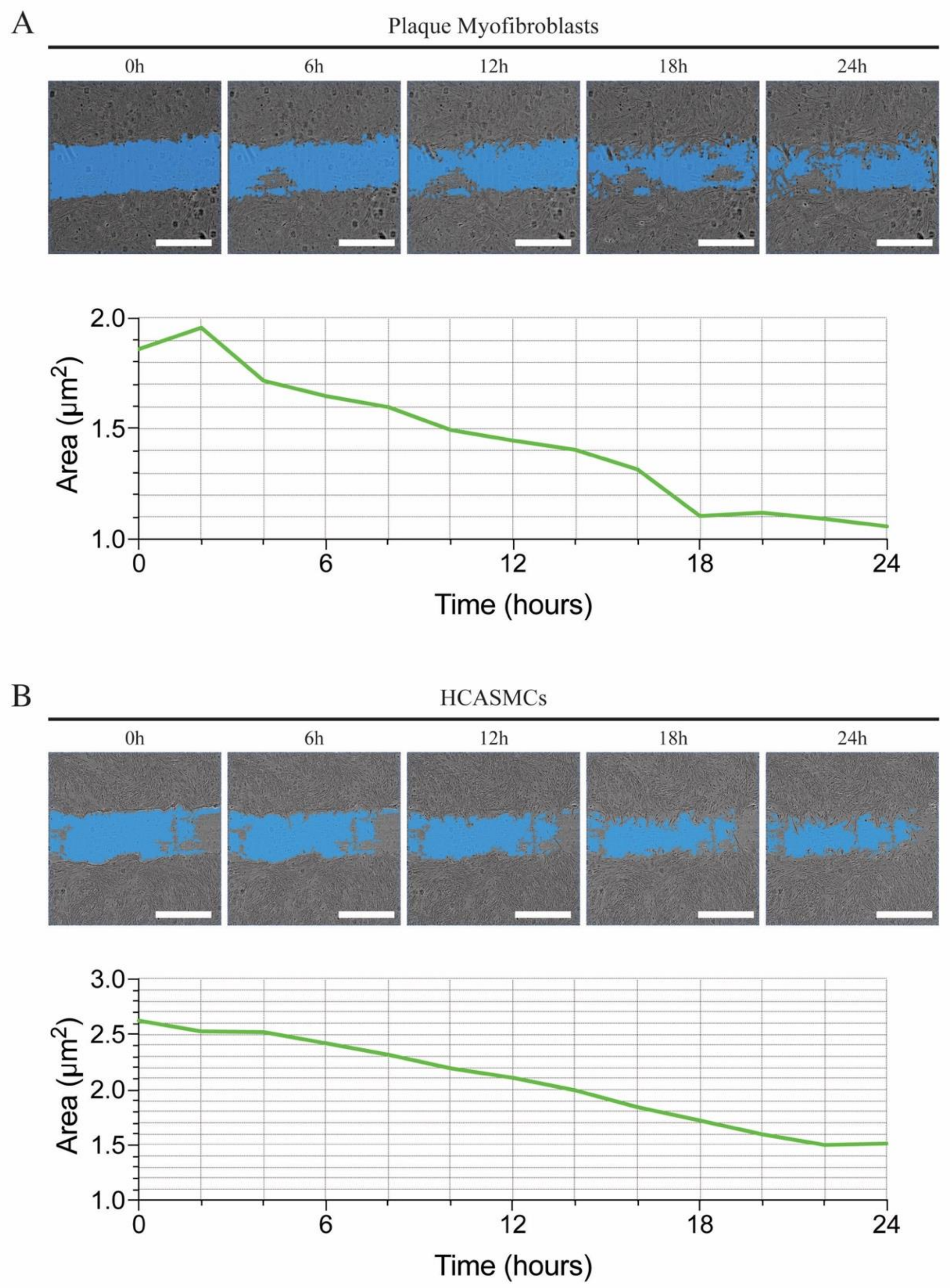

(A) Representative images and a line graph of the scratch assay performed on plaque myofibroblasts showing the migration activity and the wound size at various stages of the healing process as 0, 6, 12, 18, and 24 hours. **(B)** Representative images and a line graph of the scratch assay performed on HCASMCs show the migration activity and the wound size at various stages of the healing process as 0, 6, 12, 18, and 24 hours. Scale bars = 400 μm

Figure S3. Transcriptional differences in plaque myofibroblasts.

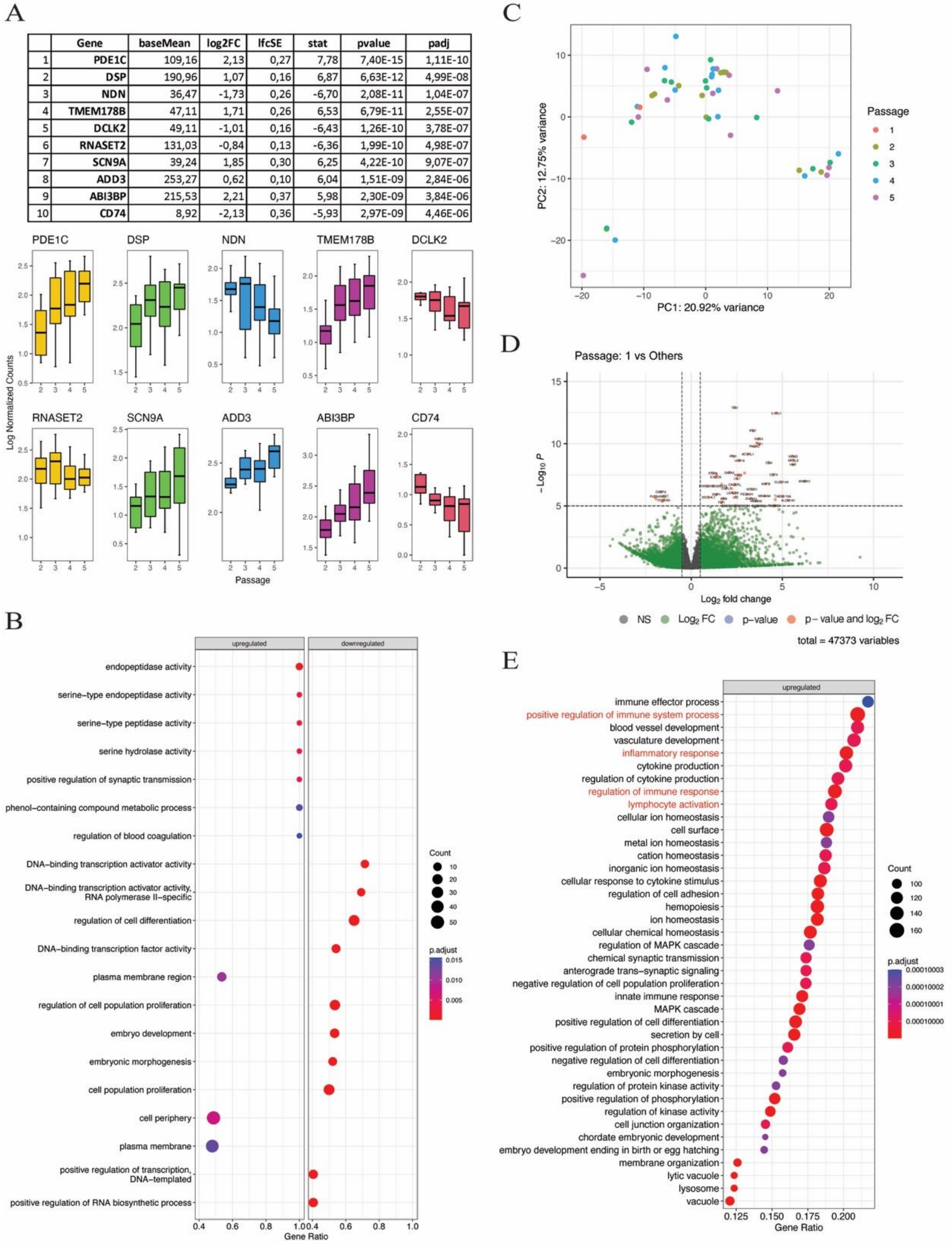

	Gene	baseMean	log2FC	lfcSE	stat	pvalue	padj
1	PDE1C	109,16	2,13	0,27	7,78	7,40E-15	1,11E-10
2	DSP	190,96	1,07	0,16	6,87	6,63E-12	4,99E-08
3	NDN	36,47	-1,73	0,26	-6,70	2,08E-11	1,04E-07
4	TMEM178B	47,11	1,71	0,26	6,53	6,79E-11	2,55E-07
5	DCLK2	49,11	-1,01	0,16	-6,43	1,26E-10	3,78E-07
6	RNASET2	131,03	-0,84	0,13	-6,36	1,99E-10	4,98E-07
7	SCN9A	39,24	1,85	0,30	6,25	4,22E-10	9,07E-07
8	ADD3	253,27	0,62	0,10	6,04	1,51E-09	2,84E-06
9	ABI3BP	215,53	2,21	0,37	5,98	2,30E-09	3,84E-06
10	CD74	8,92	-2,13	0,36	-5,93	2,97E-09	4,46E-06

(A) Top 10 time-trend genes identified performing DEG analysis among passages. (**B**) GO pathway enrichment analysis of time-trend genes in plaque myofibroblasts. **(C)** PCA plot showing that passage 1 groups far from all the other passages. **(D)** Passage 1 showed several differential expressed genes, immune cells specific, when compared to the other passages. **(E)** GO pathway enrichment analysis of DEGs in plaque myofibroblasts at passage 1.

Figure S4. Comparison between CEA- and TEA-derived plaque myofibroblasts.

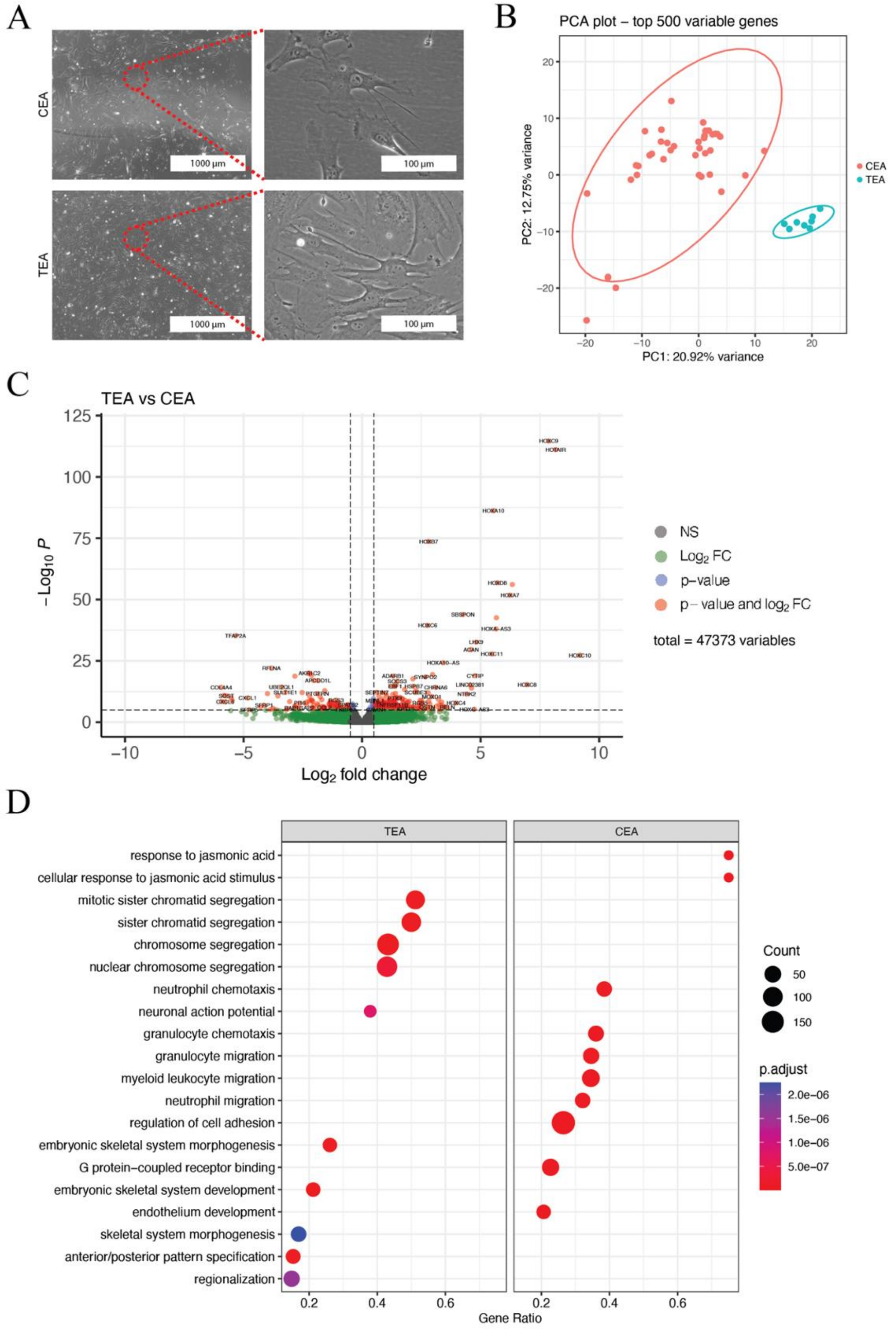

(**A**) Representative images of CEA vs TEA plaque myofibroblasts. **(B)** PCA plot of TEA- and CEA derived plaque myofibroblasts. **(B)** Differential gene expression analysis of TEA vs CEA plaque myofibroblasts. (**D**) GO pathway enrichment analysis of DEGs in TEA vs CEA plaque myofibroblasts.
